# Identification of surface proteins in *Enterococcus faecalis *V583

**DOI:** 10.1186/1471-2164-12-135

**Published:** 2011-03-01

**Authors:** Liv Anette Bøhle, Tahira Riaz, Wolfgang Egge-Jacobsen, Morten Skaugen, Øyvind L Busk, Vincent GH Eijsink, Geir Mathiesen

**Affiliations:** 1 Department of Chemistry, Biotechnology, and Food Science, The Norwegian University of Life Sciences, 1432 Ås, Norway; 2 Department of Molecular Biosciences, Glyconor Mass Spectrometry, University of Oslo, 0316 Oslo, Norway

## Abstract

**Background:**

Surface proteins are a key to a deeper understanding of the behaviour of Gram-positive bacteria interacting with the human gastro-intestinal tract. Such proteins contribute to cell wall synthesis and maintenance and are important for interactions between the bacterial cell and the human host. Since they are exposed and may play roles in pathogenicity, surface proteins are interesting targets for drug design.

**Results:**

Using methods based on proteolytic "shaving" of bacterial cells and subsequent mass spectrometry-based protein identification, we have identified surface-located proteins in *Enterococcus faecalis *V583. In total 69 unique proteins were identified, few of which have been identified and characterized previously. 33 of these proteins are predicted to be cytoplasmic, whereas the other 36 are predicted to have surface locations (31) or to be secreted (5). Lipid-anchored proteins were the most dominant among the identified surface proteins. The seemingly most abundant surface proteins included a membrane protein with a potentially shedded extracellular sulfatase domain that could act on the sulfate groups in mucin and a lipid-anchored fumarate reductase that could contribute to generation of reactive oxygen species.

**Conclusions:**

The present proteome analysis gives an experimental impression of the protein landscape on the cell surface of the pathogenic bacterium *E. faecalis*. The 36 identified secreted (5) and surface (31) proteins included several proteins involved in cell wall synthesis, pheromone-regulated processes, and transport of solutes, as well as proteins with unknown function. These proteins stand out as interesting targets for further investigation of the interaction between *E. faecalis *and its environment.

## Background

Enterococci are versatile Gram-positive bacteria that can survive under harsh conditions. Most enterococci are non-virulent and commonly found in fermented food and in the gastrointestinal (GI) tract of humans and animals. Other strains are opportunistic pathogens that contribute in a large number of nosocomial infections worldwide [1]. The mechanism underlying the switch from a harmless microbe into a life-threatening pathogen entering the host bloodstream is not well known. It is believed that the bacteria normally are well controlled in the GI tract of healthy individuals, whereas a weakened host immune system and/or development of bacterial traits to occupy new niches may lead to translocation to the bloodstream [2]. The past decade has shown a dramatic increase in antibiotic resistance of *Enterococcus *species, creating an increased need for developing new ways to combat these bacteria. To achieve this, in-depth insight in the physiology, virulence and epidemiology of enterococci is required.

*Enterococcus faecalis *is one of the most frequent *Enterococcus *species in the GI tract [3,4] and accounts for at least 60% of the bacteraemia caused by *Enterococcus *species [1]. The genome sequence of three *E. faecalis *strains (V583; [5], OG1RF [6], Symbioflor 1 [7]) have been completed, and several genome projects are ongoing. In the genome sequence of *E. faecalis *V583, a vancomycin resistant clinical isolate, over a quarter of the genome consists of mobile or foreign DNA, including pathogenicity islands. The abundance of mobile elements in *E. faecalis *probably contributes to accumulation of virulence and drug determinants. Several studies have revealed proteins that contribute to the virulence of *E. faecalis *[8-10], but it has so far not been possible to link virulence to one or very few key gene products. Since virulence depends on the ability to colonize the GI tract and interact with host cells and proteins in the GI tract, secreted proteins and proteins located on the cell surface are thought to be important. One well-studied secreted virulence factor is cytolysin, which is toxic or lytic to bacterial and human cells [9,11]. Several adhesion factors facilitating binding to mucosal and epithelial surfaces have been reported [2]. In addition to involvement in adhesion, surface proteins may affect virulence in other ways, for example by involvement in cell-cell signalling [9], interactions with the host immune system, sensing environmental factors, or protection from environmental factors.

To understand the success of bacterial pathogens and their adaption to the GI tract it is important to get an impression of the repertoire of surface associated proteins. According to the LocateP database [12], which contains genome-wide predictions for the subcellular locations of bacterial proteins, 306 proteins in *E. faecalis *are predicted to be covalently anchored to cell surface, primarily via N-terminal or lipid anchors. Another 67 are predicted to be secreted or non-covalently attached to the surface. There is only limited experimental data supporting these predicted locations [13]. Furthermore, despite their expected importance for bacterial behaviour and impact, the function of most of the predicted surface and secreted proteins remains unknown.

In the past decade, the extracellular proteomes of several Gram-positive bacteria have been analyzed using proteomics approaches. Many of these studies employed some kind of protein extraction methods from culture supernatants and/or cell wall fractions followed by two-dimensional electrophoresis and mass spectrometry-based protein identification (e.g. [14-18]). Recently, more direct and rapid methods for the "in situ" identification of surface proteins have been developed which are based on "shaving" the surface of intact bacteria with proteolytic enzymes, followed by identification of the released peptides by liquid chromatography (LC) and tandem mass spectrometry (MS/MS) [19]. An advantage of this approach is that the proteolytic enzymes will only have access to proteins that are exposed on the surface of the bacterial cell, which could limit contamination with intracellular proteins. This approach should in principle be biased towards proteins that are of particular importance for bacterial interactions with the environment. Indeed, the "shaving" approach has been applied successfully in the search for new bacterial epitopes. In a landmark study, Rodriguez-Ortega et al [19] identified in total 72 proteins in *Streptococcus pyogenes *M1_SF370 by shaving the bacterial surface with trypsin or proteinase K. The identified proteins included known protective antigens and also revealed new promising candidate antigens for vaccine development.

In the present study, we have applied the "shaving" approach to identify the surface proteome of *E. faecalis *V583. Cells were treated with free trypsin or trypsin anchored to agarose beads to shave off and digest surface-exposed proteins. Using a combination of experiments, 69 surface-located proteins were identified, including proteins assumed to be involved in pathogenicity and several proteins with unknown function. We also identified proteins that are thought to be cytoplasmic, but which tend to be found at bacterial surfaces too. We discuss the putative roles and relevance of several of the identified proteins and we compare the various approaches. The results provide a basis for the identification and further study of novel proteins putatively involved in pathogenicity and adaptability of *E. faecalis *V583.

## Methods

### Culture conditions and surface shaving

Overnight cultures of *E. feacalis *V583 [5] were diluted in fresh prewarmed brain heart infusion (BHI) medium (Oxoid Ltd., Hampshire, England) to an OD_600_~0.2 and incubated at 37°C without agitation. The bacteria were harvested on the transition between late exponential and stationary phase (OD_600_~1.7) by centrifugation (3000 × g, 10 min, 4°C). The cell pellet from 100 ml of culture was washed three times with 10 ml PBS by centrifugation (3000 × g, 10 min, 4°C) and subsequently resuspended in PBS containing 40% sucrose. Three different shaving reactions were set-up, all containing 5 mM DTT and all with the same final concentration of cells: (1) addition of 20 μg trypsin (Promega, Mannheim, Germany), (2) addition of trypsin-agarose (100 units; Invitrogen, Karlsruhe, Germany), or (3) no addition of trypsin (untreated). The samples were incubated for 1 or 2 hours at 37°C with shaking at 300 rpm. After incubation the cells were pelleted by centrifugation (3000 × g, 10 min) and the supernatants were collected for further protein digestion with 1 μg freshly added trypsin over night (16-18 h) at 37°C with agitation at 400 rpm. Cell samples taken before (i.e. after resuspending in PBS) and after the different enzymatic treatments were used to test cell viability by plating appropriate dilutions on BHI agar plates and counting of colony forming units (CFU). The overnight trypsin digestion of the supernatants was stopped by adding formic acid to a final concentration of 0.1% (v/v). Prior to nanoLC-MS/MS analysis, peptides were concentrated and purified in two steps using C_18 _Dynabeads (Invitrogen) in the first step and C18 StageTips [20] in the second step. For each treatment, samples from four biological replicates were analysed.

### Nanoflow on-line liquid chromatographic MS analysis of trypticpeptides

Reverse phase (C18) nano online liquid chromatographic MS/MS analyses of tryptic peptides were performed using a HPLC system consisting of two Agilent 1200 HPLC binary pumps (nano and capillary) with corresponding autosampler, column heater and integrated switching valve. This LC system was coupled via a nanoelectrospray ion source to a LTQ-Orbitrap mass spectrometer (Thermo Fisher Scientific, Bremen, Germany). For the analyses, the peptide solution was injected onto the 5 × 0.3-mm extraction column filled with Zorbax 300 SB-C18 of 5-μm particle size (Agilent, Waldbronn, Germany). Samples were washed with a mobile phase consisting of 97% 0.1% formic acid & 3% acetonitrile. The flow rate of 4 μl/min provided by the capillary pump. After 7 min, the switching valve of the integrated switching valve was activated, and the peptides were eluted in the back-flush mode from the extraction column onto a 150 × 0.075-mm C_18_, 3-μm resin, column (GlycproSIL C18-80Å, Glycpromass, Stove, Germany). The mobile phase consisted of acetonitrile and MS grade water, both containing 0.1% formic acid. Chromatographic separation was achieved using a binary gradient from 5 to 55% of acetonitrile in 120 min. The flow rate of 0.2 μl min^-1 ^was provided by the nanoflow pump.

Mass spectra were acquired in the positive ion mode applying a data-dependent automatic switch between survey scan and tandem mass spectra (MS/MS) acquisition. Peptide samples were analyzed by collision induced dissociation (CID) in the LTQ ion trap by acquiring one Orbitrap survey scan in the mass range of *m/z *380-2000 followed by CID of the six most intense ions in the ion trap. The target value in the LTQ-Orbitrap was 1,000,000 for survey scan at a resolution of 60,000 at *m/z *400 using lock masses for recalibration to improve the mass accuracy of precursor ions. Fragmentation was performed with a target value of 5,000 ions. The ion selection threshold was 500 counts. Selected sequenced ions were dynamically excluded for 180 s.

### MS data analysis

Mass spectrometric data were first analyzed by generating msf files from raw MS and MS/MS spectra using the Proteome Discoverer 1.0 software (Thermo Fisher Scientific) and the database searches were then performed with an in house maintained *E. faecalis *V583 protein sequence database, using the SEQUEST search engine. The following criteria were applied; database decoy, true; Enzyme name, trypsin (full); Missed cleavage sites, 2; Precursor mass tolerance, 10 ppm; fragment mass tolerance: 0.6 Da; dynamic modifications: N-term acetyl (any N-terminus), oxidation (M), carboxymethyl (C), deamidated (N, Q). Proteins were considered as significant hits if the following conditions were met: XCorr higher than 2.0; false discovery rate less than 5%; identified by at least two different peptides; identified in at least two of the independent parallels by at least one peptide in each.

### SDS-PAGE analysis

To visualize proteins or protein fragments that were resistant to trypsin, 20 μl of the supernatant from the over-night trypsination were applied to 10% NuPAGE Novex Bis-Tris gels (Invitrogen). Only samples after two-hour incubation were studied. The gels were stained using SilverSNAP Stain for Mass Spectrometry (Pierce, Rockford, IL) following the manufacturer's procedure. After the silver staining, the gel-lane was sliced into 12 pieces, and destained using the protocol included in SilverSNAP Stain for Mass Spectrometry kit. Each gel piece was then incubated with 0.1 μg trypsin in 25 μl 25 mM ammonium bicarbonate, over night at 37°C and 400 rpm. The trypsin reactions were stopped by adding 0.1% formic acid. The supernatants were transferred to new tubes, and the rest of the peptides were extracted from the gel pieces by incubating with 0.1% (v/v) trifluoroacetic acid in 60% (v/v) acetonitrile, at 37°C, 400 rpm for 10 min. The extracts from three gel-pieces were pooled together (giving four samples from each treatment). The peptides were dried in a speed-vac, and rehydrated in 30 μl 0.1% (v/v) TFA. The peptide samples were desalted using C18 stage tips [20] prior to nanoLC-MS/MS. Proteins were considered as significant hits if the following conditions were met: XCorr higher than 2.0; false discovery rate less than 5%; identified by at least two different peptides; identified in at least one of the three samples from each treatment.

### Bioinformatic analysis of protein localization

Protein sequences used for *in computo *analysis of the localization of the identified proteins were extracted from the LocateP database [12] and analyzed using several bioinformatic tools. Putative N-terminal signal sequences and cleavage sites were predicted using the Signal P 3.0 server [21] and LipoP v 1.0 [22]. The TMHMM Server v. 2.0 [23] was used to predict proteins with multiple transmembrane helices or N-terminal transmembrane anchors. Proteins with features indicating non-classical secretion were predicted using the SecretomeP 2.0 Server [24]. Domain annotations were done using Pfam [25]. After these verifications, the predicted localizations for 62 of the 69 proteins discussed below correspond to those given in the LocateP database (updated March 10, 2010). For seven proteins, we reached a different conclusion than LocateP, as described in results and discussion.

## Results and discussion

### Protein identification

Before carrying out the experiments, we performed extensive tests to find optimal conditions for the trypsin treatment. Most importantly, we checked the effect of incubation time (30 min to 24 hours) on cell viability. Incubation times of 2 hours or less did not lead to significant reductions in the CFU number (Additional file 1), whereas longer incubation times led to decreased viability (data not shown). Based on these observations, incubation times were set to one or two hours. Two hour incubations led to a higher number of identified proteins (Additional file 2). Generally, the longer incubations did not lead to an increase in the fraction of cytoplasmic proteins, confirming the absence of cell lysis during the enzymatic treatment (Additional file 2).

**Figure 1:**
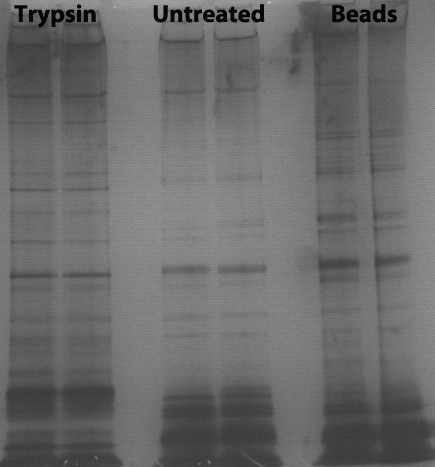
**SDS-PAGE analysis of the supernatants obtained after treating intact cells with trypsin; see materials and methods for details**. The gel shows the results from cells treated with trypsin, cells treated with trypsin beads and a control sample ("untreated") where no trypsin was added. Samples sizes represent approximately the same amount of cells in all lanes.

Intact bacterial cells were harvested and treated with either trypsin or trypsin beads (trypsin bound to agarose beads) for one or two hours. Because trypsin beads are less likely to penetrate the cell wall than free trypsin, they are more likely to only act on proteins that protrude from the cell wall. As a control, cells were incubated for one or two hours without adding trypsin. Direct analysis of released tryptic peptides led to the identification of 57 unique proteins (Table 1). Subsequent analysis of solubilized proteins and large protein fragments using SDS-PAGE followed by MS-based identification (Figure 1; see Materials and Methods) led to the identification of another 12 unique proteins (Table 2). The sequences of all identified proteins were analysed using a variety of bioinformatic tools (LocateP, SignalP, Pfam, LipoP, pSORT and TMHMM) to verify or (for EF2860, EF0071, EF0123, EF0164, EF0394, EF0417 & EF_B0004) to adjust the localization given in the LocateP database. The results are incorporated in Table 1 &2 and are discussed in appropriate sections, below.

**Table 1:** Proteins identified by LC-MS/MS analysis of tryptic fragments obtained after different treatments of intact *E. faecalis *V583.

				**Peptide hits**^**d**^						
					
Gene	**Pfam**^**a**^	**Gene product**^**b**^	**Predicted localization**^**c**^	Un-treated 1 hour	Un-treated 2 hours	Trypsin 1 hour	Trypsin 2 hours	Beads 1 hour	Beads 2 hours	Total cover-age%
EF_B0004^e^	Bacterial extracellular solute-binding proteins, family 5	TraC protein	Cell wall			2	2	(1)	(1)	13.0
EF0071	Contains trehalase domain	Putative lipoprotein	Lipid anchor SP-II, VVS-CF			(3)	2			10.0
EF0123	Contains nine Clostridial hydro-phobic W domain	Hypothetical protein	Secreted SP-I, AYA-LE	5	2	9	7	(1)	4	23.2
EF0164		Putative lipoprotein	Lipid anchor SP-II, FTS-CG	2	3	2	3		2	32.3
EF0176^f^	Basic membrane protein	Hypothetical protein	Lipid anchor SP-II, LAA-CG			3	5		2	25.7
EF0177^f^	Basic membrane protein	Hypothetical protein	Lipid anchor SP-II, LAA-CG			5	7	2		32.1
EF0195^g^		Phospho-glycerate mutase 1	Cytoplasmic			2	(1)			10.5
EF0199^g^		30S ribosomal protein S7	Cytoplasmic				2			30.1
EF0200^g^		Elongation factor G	Cytoplasmic	(1)		7	7			22.4
EF0201^fg^		Elongation factor Tu	Cytoplasmic	(1)	2	11	8	5	4	47.1
EF0205		30S ribosomal protein S10	Cytoplasmic			2			(1)	29.4
EF0206^g^		50S ribosomal protein L3	Cytoplasmic			2	5			23.4
EF0207^g^		50S ribosomal protein L4	Cytoplasmic			(1)	2			15.9
EF0211		50S ribosomal protein L22	Cytoplasmic	(1)	2		2	(1)	(1)	17.4
EF0218^g^		50S ribosomal protein L5	Cytoplasmic			(1)	3			21.2
EF0221^g^		50S ribosomal protein L6	Cytoplasmic			(1)	3			27.0
EF0223		50S ribosomal protein L18	Cytoplasmic			(1)	2			39.0
EF0226		50S ribosomal protein L15	Cytoplasmic	(1)	(2)	2	(1)	(1)	(1)	21.2
EF0228		Adenylate kinase	Cytoplasmic			2	2			19.4
EF0234^g^		50S ribosomal protein L17	Cytoplasmic			2	(1)			26.0
EF0304	-	Putative lipoprotein	Lipid anchor SP-II, LSA-CS			(1)	2		2	20.5
EF0394	Cysteine-rich secretory protein family	Secreted antigen, putative	Secreted SP-I, ALA-DN	2				3	(1)	17.8
EF0417	-	Hypothetical protein	Secreted SP-I, VNA-LN	3	2	4	4		3	11.2
EF0502	-	Hypothetical protein	Multiple transmembrane proteins						2	7.9
EF0633		tyrosyl-tRNA synthetase	Cytoplasmic				2			6.9
EF0685^f^		Rotamase family protein	Lipid anchor SP-II, LAA-CS				2			10.8
EF0737		Amidase	N-terminal anchor				2			6.8
EF0907^f^	-	Peptide ABC transporter peptide-binding protein	Lipid anchor SP-II, LAA-CG				2			16.4
EF0916		50S ribosomal protein L20	Cytoplasmic	(1)	(1)	2				27.7
EF0970^e^		50S ribosomal protein L27	Cytoplasmic			2	2			34.7
EF0991		Penicillin-binding protein C	N-terminal anchor			(1)	2			11.2
EF1033		Lipoamidase	Cell wall anchored LPxTG SP-I, AQE-SI			2	2			4.7
EF1046^g^		Pyruvate kinase	Cytoplasmic	(2)		(2)	2			11.6
EF1167^g^		Fructose-bisphosphate aldolase	Cytoplasmic	(1)	(2)	2	4	3	(1)	30.8
EF1264^f^		Sulfatase domain-containing protein	Multiple trans-membrane proteins	2		10	11	(1)	5	29.8
EF1308^fg^		Dnak protein	Cytoplasmic	(1)		2	3			23.7
EF1319	-	Hypothetical protein	N-terminal anchor			(1)	3			23.3
EF1379		Alanyl-tRNA synthetase	Cytoplasmic					2		3.3
EF1420	-	Hypothetical protein	Lipid anchor SP-II, MTA-CS			2	(1)			15.6
EF1523^e^	-	Hypothetical protein	Cytoplasmic			(1)	3			6.4
EF1613^fg^		Formate acetyltransferase	Cytoplasmic			3	(2)			13.4
EF1818^f^		Coccolysin	Secreted SP-I, VAA-EE	8	3	8	5	(1)	2	45.1
EF1898		50S ribosomal protein L19	Cytoplasmic			(1)	2			30.4
EF1961^fg^		Enolase	Cytoplasmic			2	4	(1)		25.7
EF1964^fg^		Glycer-aldehyde-3-phosphate dehydrogenase (GAPDH)	Cytoplasmic			3	8			36.0
EF2144	-	Putative lipoprotein	Lipid anchor SP-II LTA-CS	(1)	2	(1)	2			22.4
EF2174	Glycosyl hydrolases family 25	Hypothetical protein	Secreted SP-I, ASG-EE				3			4.5
EF2398^g^		30S ribosomal protein S2	Cytoplasmic	(1)		2	4	2	(1)	19.2
EF2556^f^		Fumarate reductase flavoprotein subunit	Lipid anchor SP-II, ATG-CT	40	39	40	41	22	37	79.2
EF2718^ge^		50S ribosomal protein L1	Cytoplasmic			2	2			24.0
EF2746		DltD protein	N-terminal anchor	(1)	2	2	3	(1)	2	10.4
EF2860^f^		YkuD putative, peptido-glycan binding domain	Cell wall, SP-I VYF-QS,	2	6	5	9	(1)	7	36.3
EF2864	-	Hypothetical protein	Lipid anchor SP-II, LTA-CR	2	2	3	4	2	2	25.7
EF2925^g^		Cold-shock domain-contain protein	Cytoplasmic	(1)	2			(1)	2	45.5
EF3041^f^		Pheromone binding protein	Lipid anchor SP-II, LAA-CG	2	3	2	7		2	24.3
EF3256^f^		Pheromone cAD1 precursor lipoprotein	Lipid anchor SP-II, LAA-CG			(1)	2			19.4
EFA0003		TraC protein	Lipid anchor SP-II, LGA-CN				2			9.4

Further details on the Sequest-based protein identification process are provided in the Materials and Methods section and in Table S5 (Additional file 4).

^a^Significant hits (or absence thereof, indicated by -) obtained after searches in Pfam [25] for putative and hypothetical proteins.

^b^All data extracted from the LocateP database [12] with two exceptions: EF1033 (annotated as 6-aminohexanoate-cyclic-dimer hydrolase; annotated as putative, in LocateP) and EF2860 annotated as ErfK/YbiS/YcfS/YnhG family protein; annotated as putative, in LocateP).

^c^Predicted localization and potential cleavage site. Localization is based on LocateP annotations, with seven exceptions (for Tables 1 and 2 in total) that are all explicitly mentioned in the text. See also Table 3.

^d^The column shows the number of peptide hits from four biological replicates. Protein identifications were considered significant using the criteria described in Materials and Methods. One criterium was the detection of at least two different peptides; another the detection of peptides in at least two independent parallels; these criteria are met for all listed proteins. Putative, non-significant additional identifications of these proteins (based on just one peptide and/or on just one parallel) are indicated in parentheses.

^e^SecretomeP value >0.5; this means that the protein is predicted to be secreted via a "non-classical" pathway.

^f^Proteins that have been identified as being localised on the surface in a previous study of *E. faecelis *JH2-2 [13].

^g^Cytoplasmic proteins that have been identified in other studies of the surface proteomes of Gram-positive bacteria. See text for references.

**Table 2:** Additional proteins identified using the SDS-PAGE approach after different treatments.

				**Peptide hits**^**d**^			
					
Gene	**Pfam**^**a**^	**Gene products**^**b**^	**Predicted localization**^**c**^	Untreated 2 hours	Trypsin 2 hours	Beads 2 hours	Coverage%
EF0517^f^		2-dehydropantoate 2-reductase	Cytoplasmic			2	13.1
EF0968^f^		50S ribosomal protein L21	Cytoplasmic			2	35.3
EF2221		ABC transporter. substrate-binding protein	Lipid anchor SP-II, LSA-CG			2	8.8
EF2224	Four DUF11 repeats	Cell wall surface anchor family protein	Cell wall, LPxTG, SP-I, MNA-FA			2	1.4
EF2633^f^		Chaperonin. GroEL	Cytoplasmic			4	9.2
EF2713	Gram positive anchor	Cell wall surface anchor family protein	Cell wall, LPxTG, SP-I VWA-ED		2		9.4
EF2715^f^		Ribosomal protein L7/L12	Cytoplasmic			2	29.5
EF2857^e^		Penicillin-binding protein 2B	N-terminal anchor			3	4.8
EF2903^e^		ABC transporter, substrate-binding protein	Lipid anchor SP-II, LGA-CG		6	2	20.2
EF3106^e^		Peptide ABC transporter. peptide-binding protein	Lipid anchor SP-II, LAA-CG		2		3.4
EF3257		Pyridine nucleotide-disulfide family oxidoreductase	Multiple transmembrane proteins		2		7.3
EFA0052		Surface exclusion protein Sea1	Cell wall LPxTG SP-1, VQA-AE	2	2	2	6.3

The gel approach yielded 25 unique proteins in total but only 12 of these were novel compared to the list of Table 1; see Additional file 2 for more details. Further details on the Sequest-based protein identification process are provided in the Materials and Methods section and in Table S6 (Additional file 5).

^a^Significant hits obtained after searches in Pfam [25] for putative and hypothetical proteins.

^b^Data extracted from the LocateP database [12].

^c^Predicted localization and potential cleavage site. Localization is based on LocateP annotations, with seven exceptions (for Tables 1 and 2 in total) that are all explicitly mentioned in the text. See also Table 3.

^d^Number of peptide hits in each of the three treatments. Protein identifications were considered significant using the criteria described in Materials and Methods. Proteins were only considered a significant hit if at least two unique peptides were found.

^e ^Proteins that have been identified as being localised on the surface in a previous study of *E. faecelis *JH2-2 [13].

^f^Cytoplasmic proteins that have been identified in other studies of the surface proteomes of Gram-positive bacteria. See text for references.

Analysis with Signal P 3.0 [21] suggested the presence of a signal peptidase I (SPase I) cleavage site in ten of the identified proteins. Four of these proteins contained a putative C-terminal LPxTG motif, whereas one additional protein (EF2860) is likely to be cell-wall anchored because it contains a putative peptidoglycan binding domain (Pfam PF12229). It should be noted that the sequences deposited in the GenBank database for two of the four LPxTG-containing proteins (EF1033 and EF2713) lack a predicted N-terminal signal sequence. A closer look at the upstream sequences showed that the start codons probably are located 72 and 96 nucleotides upstream of the start codon suggested in the GenBank entries, for EF1033 and EF2713, respectively (Additional file 3). After this N-terminal "extension" SignalP and LipoP detected a putative SPase I cleavage site in both sequences.

**Figure 2:**
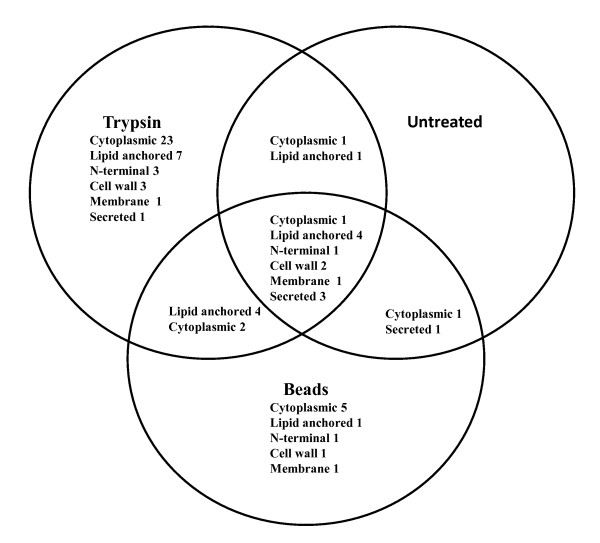
**Overview over the identifcation of 69 surface-located proteins using various treatments of enterococcal cells**. The putative cellular localizations of the identified proteins, as provided in Table 3 are indicated. In total 58, 16 and 29 proteins were identified in the trypsin, untreated and beads samples, respectively. Note that this figure combines the data from the one and two hour treatments and includes the data from both the direct LC-MS/MS analysis and the SDS-PAGE-based approach. More details about the numbers of identified proteins in the various experiments are provided in Additional file 2. Proteins containing multiple transmembrane helices are indicated by "Membrane"; proteins indicated by "Cell Wall" include 4 proteins with LPxTG anchors and two proteins containing domains known to display cell wall binding affinity.

Table 3 gives an overview over the predicted localizations of the 69 identified unique proteins and shows that the methods yielded a strong bias towards identifying proteins that are predicted to be covalently anchored to the cell wall or to carry lipid anchors. Several proteins were identified in more than one experiment and an overview is provided in Figure 2. Treatment with free trypsin yielded 58 proteins and treatment with trypsin beads yielded 29 proteins. Analysis of samples from untreated cells yielded 16 proteins. More detailed information concerning the numbers of proteins identified after the various treatments is provided in additional file 2. Details of the proteomic analysis are provided in Additional file 4 and Additional file 5 containing Tables S5 and S6, respectively.

**Table 3:** Summary of the identified proteins grouped according to predicted localization.

**Predicated localization**^***a***^	Number of identified unique proteins	**Number in the *E. faecalis *V583 genome**^***b***^	Percent identified
Cytoplasmic	33	2303	1.4
Membrane^c^	3	588	0.5
Lipid anchor	17	74	23.0
N-terminal anchor	5	190	2.6
LPxTG proteins	4	38	10.5
Cell wall associated	2	ND^d^	ND^d^
Secreted	5	59	8.5
C-terminal anchor	0	4	0
Secreted via minor pathway	0	8	0
Sum	69	3264	2.1

^*a *^Localization data are from LocateP (Zhou et al., 2008), with the seven corrections described in the text. According to Locate P, the numbers of identified cytoplasmic, lipid anchor, N-terminal anchor, cell wall associated and secreted proteins.

^b ^Data from the LocateP database.

^c ^Containing multiple transmembrane helices.

^d ^Not detected (ND), LocateP only predicts cell wall proteins with LPxTG motifs.

The number of proteins only identified after a "shaving" treatment amounted to 53. Nine of these were only found after treatment with trypsin beads, 38 were only found after treatment with free trypsin and six were found after both treatments (Figure 2). The 15 proteins identified with only trypsin-beads or with both trypsin and trypsin beads are likely to be exposed on the surface of the cell wall, whereas the 38 unique proteins found in the free trypsin samples fraction are probably localized deeper in the cell wall.

**Figure 3:**
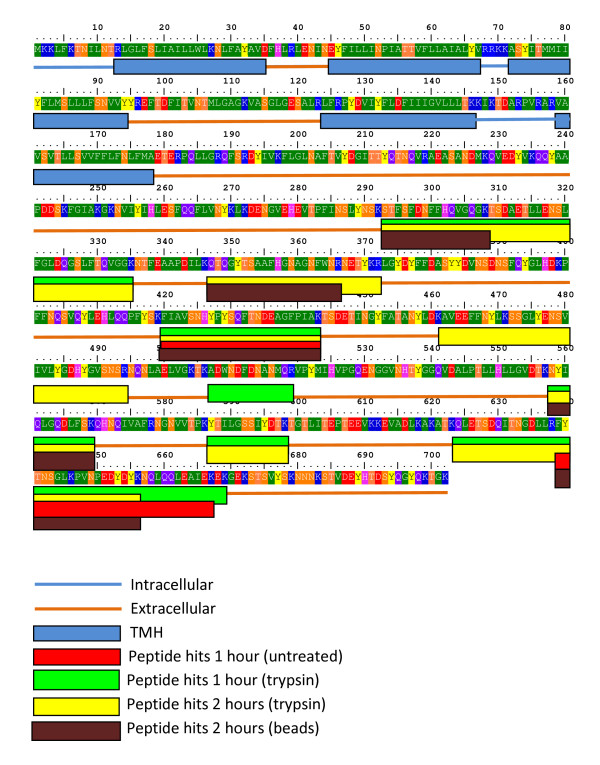
**Amino acid sequence and predicted topology of EF1264**. The predicted trans-memberane helices (TMH) and the identified tryptic peptides after different treatments are mapped on the sequence, using the color coding indicated in the figure.

Of the 16 proteins identified from untreated cells, 12 were identified in all three treatments (Figure 2). While three of these are predicted to be secreted and one (EF0201) is probably cytoplasmic, the others are predicted to be attached to the bacterial surface through an anchor (five lipo-anchors and one LPxTG anchor) or cell wall binding domain (one, EF2860) or even as integral membrane protein (one, EF1264). The trypsin-independent release of these proteins may be a result of natural shedding, a phenomenon that indeed has been observed previously, in particular for lipoproteins [15,26]. According to a TMHMM topology prediction EF1264, annotated as membrane protein, contains five N-terminal transmembrane helices and a huge extracellular domain with putative sulfatase activity of 523 residues (starting at amino acid 179). EF1264 was identified by many significant peptide hits spread over all treatments. Figure 3 shows that all identified peptides stem from the extracellular domain and that there is a 115 amino acid gap between the predicted integral membrane domain and the first identified tryptic fragment. Perhaps the extracellular domain is shedded after natural cleavage of EF1264. It is conceivable that such (apparently rather abundant) shedding is a physiologically relevant phenomenon since the sulfatase may remove sulphate from mucin, which would allow more easy degradation of mucin by glycosidases [27] and perhaps also could facilitate bacterial adhesion.

### Cytoplasmic proteins

According to the LocateP database 34 of the 69 identified proteins lack an N-terminal signal sequence and are therefore predicted to be cytoplasmic proteins. All these proteins were analysed using PSORTb v.3.0 [28] and the SecretomeP (SecP) 2.0 Server [24,29]. SecP predict proteins that are putatively secreted without having a detectable N-terminal signal sequence, i.e. by "non-classical secretion". Analysis with SecP indicated that four of the proteins predicted to be cytoplasmic (EF0970, EF1523, EF2718 and EF_B0004) may follow non-classical secretion. PSORTb predicted EF_B0004 (TraC protein) to be a cell wall protein and Pfam gave a significant hit against "Bacterial extracellular solute-binding proteins, family 5" (PF00496). It has been shown that TraC proteins play a role as surface pheromone receptor and are thereby involved in the regulation of the conjugation process [30,31]. Therefore, EF_B0004 was classified as a cell wall protein in this study. The other three proteins were retained as cytoplasmic, despite the fact that PSORTb predicted EF0970 (ribosomal protein L27) to have an extracellular location.

Identification of cytoplasmic proteins at extracellular locations is not unusual and at least 20 of the 33 proteins found in this study have been identified in previous studies of the secretomes or surface proteomes of Gram-positive bacteria (Tables 1 and 2) [13,18,26,32-36]. The majority of the identified cytoplasmic proteins were unique to the trypsin fraction and/or the beads fraction, indicating that these proteins bind to the cell envelope and need to be "shaved" from the surface, despite the lack of known binding motifs or domains. Many of the identified cytoplasmic proteins are highly abundant proteins like ribosomal proteins (Rbps; more than 20% of all identified proteins), EF-Tu/G, GADPH and chaperones, which suggests that cell lysis rather than an unknown active secretion process determines their extracellular presence. While the cell viability checks described above indicate that cell lysis due to the trypsin treatment is unlikely, it is conceivable that cell lysis in the cell culture prior to the trypsin treatment may have released intracellular proteins that somehow have re-associated with the cell envelope and escaped proteolytic degradation [26,37].

While cytoplasmic proteins found in studies such as the present generally must be considered contaminants, there have been speculations in the literature that some of these actually may have extracellular functions. One example is Rbp L7/L12 (EF2715) which has been identified at the surface of several Gram positive bacteria [19,26,34,35,38]. We found Rbp L7/L12 in the beads fraction only and this implies an exposed localization, similar to the localization suggested in *B. subtilis *[26]. Bacterial Rbp L7/L12 has immunogenic properties in humans [38,39] and is being explored as candidate antigen for vaccine purposes [40,41]. Recent data showed that exposure of bacterial Rbp L7/L12 is a risk factor for colorectal cancer and stimulates progression of adenomas into carcinomas [42]. There has also been some speculation about possible adhesive roles of extracellular EF-Tu, DnaK, enolase and GAPDH, all identified in the present study and in a recent study of the laboratory strain *Enterococcus faecalis *JH2-2 [13], since these proteins bind strongly to human plasminogen [32].

### Secreted proteins

Proteins were annotated as being secreted to the culture medium if the bioinformatic analyses showed the presence of a SPaseI cleavage site and did not reveal any sequence or domain known to be involved in covalent or non-covalent binding to the cell wall. Only five such proteins were identified indicating that few secreted proteins are closely associated to the microbe. Three of the secreted proteins (EF0123, EF0394, EF0417) are annotated as N-terminally anchored in the LocateP database. It is, however, not easy to differentiate between secreted proteins with processed signal peptides and proteins that retain their signal peptides as N-terminal membrane anchors [12]. The Signal P server predicted all three proteins to contain a unique cleavage site, when using both algorithms in the program. All three were detected even without trypsin treatment, suggesting a loose association with the cell envelope. Taken together, we conclude that EF0123, EF0394 and EF0417 are secreted proteins.

EF2174 was detected after trypsin treatment only and putatively encodes a glycoside hydrolase belonging to family GH25 [43]. This family comprises enzymes with lysozyme activity that are thought to be involved in peptidoglycan remodelling during cell division [44].

The well known secreted metalloprotease coccolysin, a gelatinase (EF1818; GelE) was identified after all treatments (Table 1), indicating that GelE is loosely associated to the cell surface. Coccolysin is known to be associated with virulence and is capable of degrading cellular tissues during infection, cleaving substrates such as haemoglobin, collagen and fibrin [45,46]. There are also indications that GelE is required for biofilm formation [47].

### Proteins on the surface

Among the 31 non-secreted non-cytoplasmic proteins found in this study (Table 3), three are annotated as integral membrane proteins. We identified only 0.5% of the predicted transmembrane proteins in the genome of *E. faecalis *V583 (Table 3). Such low numbers of identified transmembrane proteins are not unusual [19,26,37,48] and are likely to be due to limited accessibility of the proteins and/or a limited ability of trypsin to penetrate the cell wall. One of these transmembrane proteins, the sulfatase (EF1264), was detected by many peptides after all treatments (see above and Figure 3). The other two were only detected after trypsin treatment and only by a minimal number of peptides. EF0502 is a 781-residue hypothetical protein which according to topology predictions contains several extracellular domains. Both detected peptides are predicted to have an extracellular location. EF3257 is a 648-residue oxidoreductase belonging to the pyridine nucleotide-disulfide family with probably only two trans-membrane helices and a large extracellular domain. Both detected peptides stem from extracellular domains.

Five identified proteins are thought to be N-terminally anchored to the cell membrane via a Sec-type signal peptide that is not cleaved off during secretion. Relatively few such proteins were identified (Table 3), suggesting that they are expressed at low levels or that they generally have low accessibility for trypsin. Among these proteins are two penicillin-binding proteins (EF2857 & EF0991), one amidase (EF0737) and one protein of unknown function (EF1319). EF2857 and EF0991 are class B penicillin binding proteins (PBP), which are transpeptidases involved in the final stage of cell wall synthesis [49]. *E. faecalis *has three class B PBPs that have low affinity for β-lactams and can take over the transpeptidase activity of more high affinity PBPs when these are inhibited by antibiotics [50]. We also identified a L,D-transpeptidase (EF2860; YkuD domain), in all treatments and with a high number of peptide hits, indicating that this protein is abundant at the surface of V583. Studies on *E. faecium *have indicated that L,D-transpeptidase activity may represent another way to bypass inhibition of PBPs [51,52].

The fifth protein, DltD (EF2746), is also involved in the biosynthesis of surface structures. The *dltD *gene is part of an operon consisting four genes (*dltA-dltD*) whose gene products are all necessary to incorporate D-alanyl residues into lipoteichoic acids (LTA) [53,54]. It has previously been shown that disruption of genes in the *dlt *operon of *E. faecalis *lead to diminished adhesion to eukaryotic cells and less biofilm formation, indicating that the *dlt *operon is involved in pathogenicity [55]. Interestingly, it is not fully established whether the N-terminal anchor tethers DltD to the inner or outer leaflet of the membrane [53,56]. The fact that DltD was detected after all types of treatments may be taken to suggest that the protein is attached to the outer leaflet, as one would expect for proteins using a Sec-type signal peptide as membrane anchor.

Of the four proteins containing a putative LPxTG anchor, two (EF2224 & EF2713) have unknown functions. EF2713 is up-regulated when *E. faecalis *V583 is grown in the presence of blood [57], indicating a putative role of this protein in infection processes. The LPxTG anchor protein EF2224 contains five copies of a so-called DUF11 repeat with unknown function and is a putative member of the MSCRAMM (mirobial surface component recognizing adhesive matrix molecules) family of proteins [58]. These proteins contain tandemly repeated immunoglobulin-like folds as observed for staphylococcal adhesins. The *E. faecalis *V583 genome contains seventeen proteins belonging to the MSCRAMM family [59]. Interestingly, EF2224 is highly expressed during the infection process in humans [59].

The LPxTG anchor protein EF1033 is a lipoamidase (Lpa) cleaving lipoic acids from lipoylated molecules [60,61]. EF1033 was only detected after trypsin treatment, indicative of covalent cell wall attachment. Lipoic acid is an essential sulphur containing cofactor of several enzymes. Interestingly, so far, *E. faecalis *is the only bacterial species in which Lpa activity has been detected [61]. Jiang and Cronan [61] speculate that the Lpa is a cytoplasmic salvage enzyme, but our experimental and bioinformatic results indicate that the enzyme is a cell wall anchored protein. Most likely, Lpa recruits its substrates from the environment, such as the GI tract.

The fourth LPxTG protein is the plasmid encoded surface exclusion factor Sea1 (EFA0052) which is involved in the regulation of sex pheromone-controlled conjugation [62].

EF2860 is linked to the cell wall by a peptidoglucan binding domain and encodes a cell wall modifying transpeptidase homologous with YkuD from *Bacillus subtilis*. This protein was found after all three treatments and identified with relatively many peptide hits (Table 1), indicating that EF2860 is abundant on the surface and may show a relatively large extent of shedding. This protein may contribute to the antibiotic resistance of *E. faecalis*, as discussed above.

The most populated group of proteins identified in this study are the lipoproteins, of which 17 were detected, representing 23% of the lipoproteins putatively encoded on the *E. faecalis *genome (Table 3). Twelve of these were detected only after a "shaving" treatment. Seven of the detected lipoproteins are proteins with no predicted function. Two of these unknown lipoproteins (EF0176 & EF0177) are located on the same operon, share 70% sequence identity, and contain a "Basic membrane protein" domain (PF02608) belonging to Clan CL0144 in the Pfam database. This clan consists of proteins that are involved in chemotaxis and membrane transport of sugars as well as outer membrane proteins that are known for their antigenicity in pathogenic bacteria. Both proteins are homologous with a CD4+ T-cell-stimulating antigen in *Listeria *[63]. One of the other proteins with unknown function, EF0164, is annotated as N-terminally anchored in the LocateP database, but is classified as a lipoanchored protein on the basis of our analyses with LipoP.

Of the ten lipoproteins with predicted functions, four lipoproteins resemble the substrate-binding domains of multi-component ABC transporters for the import of peptides (EF0907, EF3106) or sugars (EF2221, EF2903). Three proteins (EF3041, EF3256, EFA0003) are involved in pheromone-regulated processes that include conjugation [31], adding to the two cell wall associated proteins involved in these processes that are discussed above (EFB0004 & EFA0052). EF0071 seems to encode a glycoside hydrolase belonging to clan GH-G in the CAZy database. The protein is classified as N-terminally anchored in the LocateP database, but our analyses with the LipoP program clearly indicated that EF0071 is a lipoanchored protein. EF0685 belongs to the rotamase family and is thus likely to be involved in extracellular protein folding, possibly by exerting prolyl-peptidyl isomerase activity.

The final lipo-anchored protein is EF2556, fumarate reductase, which was detected by remarkably large numbers of peptides after all treatments (Table 1). *E. faecalis *is one of few bacteria that produces substantial amounts of extracellular superoxide. Fumarate reductase is likely to be involved in superoxide production and may thus be an important source of oxidative stress for the host [64]. It has been demonstrated that the superoxide from *E. faecalis *promotes chromosomal instability in mammalian cells and that this can lead to colorectal cancer [65,66].

## Conclusions

In recent years, several analyses of bacterial surface proteomes have been described. Despite the improvements in the mass spectrometry methods, the numbers of identified proteins are normally in the order of 30 - 80, meaning that only a minority of the putative surface-located proteins is being found. In this type of studies, it is common to find a significant fraction of proteins that are thought to be cytoplasmic and there is some evidence that this is not just the result from artefacts such as cell lysis. We show that the large majority of the identified cytoplasmic proteins are only found after treatment with trypsin. This is an important observation, since it shows that these proteins bind tightly to the cell envelope.

In a recent published proteomics-based analysis 38 proteins were identified on the surface of *E. faecalis *JH2-2 [13]. Seventeen of these proteins were found after using a method similar to the one used here (i.e. surface shaving with trypsin) and seven of these were also found in the present study (EF0177, EF0201, EF0907, EF1613, EF1964, EF2556 and EF3256). Disparities between this type of studies may be due to many factors, e.g. differences between the strains and growth conditions or differences in the confidence of protein identification. Benachour et al [13] allowed protein identification on the basis of only one peptide hit, while we required at least two peptides for confident identification. Large inter-strain variation has been observed in several previous studies, both for secreted proteins and for proteins detected by a trypsin-shaving approach [34,67,68].

In conclusion, our studies reveal 69 surface-located proteins in *E. faecalis *V583 with varying roles in bacterial behaviour. Several of the identified proteins are involved in cell wall synthesis and maintenance as well as in cell-cell communication and seem interesting targets for drug design. We detected only a few proteins with known or conceivable functions in adhesion, but such proteins may be among the many identified proteins with unknown function. Clearly, the identified proteins with unknown function stand out as targets for more in-depth investigations and several of these are currently subjected to knock-out studies in our laboratory.

## Authors' contributions

LAB, GM, TR and VE developed the initial concept for this study. All authors participated in experimental design and coordination of the study. LAB and TR carried out shaving experiments. MS, WEJ and ØB contributed to the experimental design of the mass spectrometry experiments. LAB, TR, WEJ carried out the LC-MS/MS analysis. GM and LAB did the bioinformatics analysis. LAB, GM and VE drafted the paper, implementing contributions from all other authors. All authors read, corrected and approved the final manuscript.

## Supplementary Material

Additional file 1 **Figure S1: Control of viability of the cells before and after incubation for one or two hours with trypsin, trypsin beads or without any enzyme**. Click here for file

Additional file 2 **Table S1-S4: Number of identified proteins in each treatment PDF**. Click here for file

Additional file 3 **Figure S2: Nucleotide and amino acid sequences of EF1033 and EF2713 after adjustment of the start codon**. Click here for file

Additional file 4 **Table S5: Proteome data of the proteins identified by LC-MS analysis after different treatments**. Click here for file

Additional file 5 **Table S6: Proteome data of the proteins identified using the SDS-PAGE approach after different treatments**. Click here for file
