# Supplementary material for: Identification of surface proteins in Enterococcus faecalis V583
Source: BMC Genomics. 2011 Mar 1;12:135. doi: 10.1186/1471-2164-12-135 (PMC3059304; doi:10.1186/1471-2164-12-135)
Supplement: Additional file 1 — Figure S1: Control of viability of the cells before and after incubation for one or two hours with trypsin, trypsin beads or without any enzyme. [file 1471-2164-12-135-S1.PPT]

## Slide 1
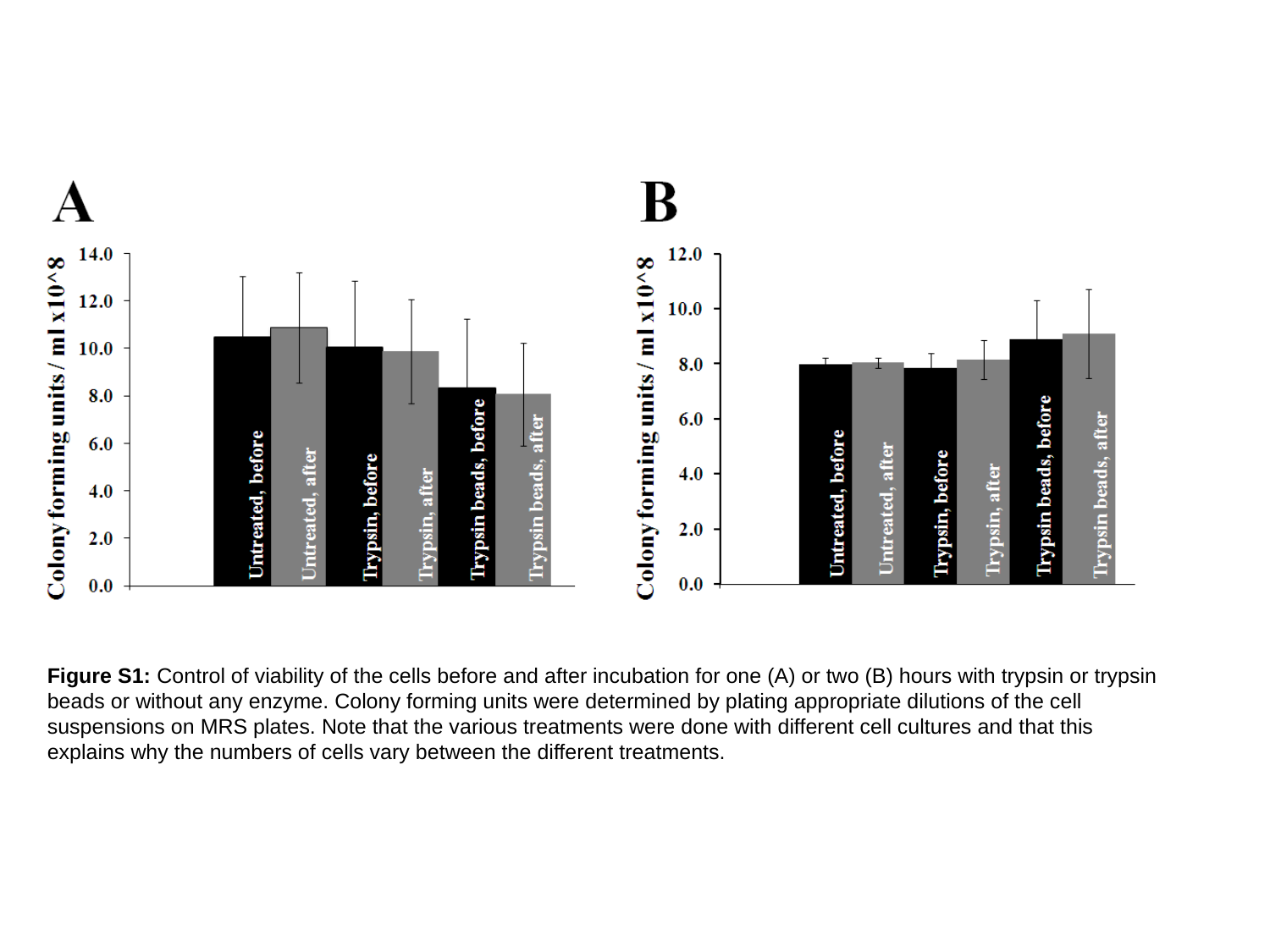

Figure S1: Control of viability of the cells before and after incubation for one (A) or two (B) hours with trypsin or trypsin beads or without any enzyme. Colony forming units were determined by plating appropriate dilutions of the cell suspensions on MRS plates. Note that the various treatments were done with different cell cultures and that this explains why the numbers of cells vary between the different treatments.
