# Supplementary material for: Identification of surface proteins in Enterococcus faecalis V583
Source: BMC Genomics. 2011 Mar 1;12:135. doi: 10.1186/1471-2164-12-135 (PMC3059304; doi:10.1186/1471-2164-12-135)
Supplement: Additional file 2 — Table S1-S4: Number of identified proteins in each treatment PDF. [file 1471-2164-12-135-S2.PDF]

Table S1. Proteins identified in the untreated fractions after one or two hours, grouped according to predicted localization.

Protein class	1 hour	2 hours	Total unique proteins
Cytoplasmic	0	3	3
Membrane[a]	1	0	1
N-terminal anchor	0	1	1
Lipid anchor	4	5	5
Cell wall	1	1	1
Secreted	4	3	4
Sum	10	13	15

[a]Containing multiple transmembrane helices

Table S2. Proteins identified after treating with free trypsin for one or two hours, grouped according to predicted localization.

Protein class	1 hour	2 hours	Total unique proteins
Cytoplasmic	17	21	27
Membrane[a]	1	1	1
Lipid anchor	8	13	14
N-terminal anchor	1	4	4
Cell wall	3	3	3
Secreted	3	4	4
Sum	33	46	53

[a]Containing multiple transmembrane helices

Table S3. Proteins identified after treating with trypsin beads for one or two hours, grouped according to predicted localization.

Protein class	1 hour	2 hours	Total unique proteins
Cytoplasmic	4	2	5
[a]Membrane	0	2	2
Lipid anchor	3	6	7
N-terminal anchor	0	1	1
Cell wall	0	1	1
Secreted	1	3	4
Sum	8	15	20

[a]Containing multiple transmembrane helices

Table S4. Proteins identified via the SDS-PAGE approach. This approach was only used for samples obtained after two hours of incubation.

Protein class	No trypsin	Free trypsin	Trypsin-beads	Total unique proteins from gel approach	Novel compared to direct LC-MS/MS
Cytoplasmic	2	4	7	9	4
MTH	0	1	0	1	1
Lipid anchor	3	7	6	8	3
N-terminal anchor	0	0	1	1	1
Cell wall	1	2	2	3	3
Secreted	3	0	2	3	0
Sum	9	14	18	25	12
Only found via gels	1	5	9	12	

[a]Containing multiple transmembrane helices
