# Supplementary material for: Identification of surface proteins in Enterococcus faecalis V583
Source: BMC Genomics. 2011 Mar 1;12:135. doi: 10.1186/1471-2164-12-135 (PMC3059304; doi:10.1186/1471-2164-12-135)
Supplement: Additional file 3 — Figure S2: Nucleotide and amino acid sequences of EF1033 and EF2713 after adjustment of the start codon. [file 1471-2164-12-135-S3.PDF]

EF1033

```
   1 CTGTATTAAT AGATAAAGGA TGTGACTTAT GAAGAAGTCG ATTTTGTTCA
                                   M  K  K  S   I  L  F  K
  51 AAAAGTTAGG GATTATTTTA TTAATTAGCC AAACACTGGT AGGGGTACCA
       K  L  G   I  I  L   L  I  S  Q   T  L  V   G  V  P
 101 ATGTTGGCAC AAGAAAGTAT ACTAGAAACA ACCGTTCAAA CAGAGACGGA
     M  L  A  Q   E  S  I   L  E  T   T  V  Q  T   E  T  E
 151 ATCAGTAACA ACACAAACCA GTCAGACTGT AGCTAACTTG GAATCTGAAA
       S  V  T   T  E  T  S   Q  T  V   A  N  L   E  S  E  T
 201 CTACTAGCCA AACGGTGATG CAGGAAAAAG AATCCTCTTC GGCAATCGCC
       T  S  Q   T  V  M   Q  E  K  E   S  S  S   A  I  A
 251 GAAAGCAGTA GCGGAAATGC GGTTGCAGTA ACTACTGAAA CCACAAATGA
     E  S  S  S   G  N  A   V  A  V   T  T  E  T   T  N  E
-----------------------------------------------------------
2101 TCAGGGAACC AAATCAACAG AAAGTACTCA TGCTTTTTCT GATAAAAATA
       Q  G  T   K  S  T  E   S  T  H   A  F  S   D  K  N  M
2151 TGATTGGGAA AAAAGAACAG CTTCCCAAAA AAGTATTACC AAAAGCAGGT
       I  G  K   K  E  Q   L  P  K  K   V  L  P   K  A  G
2201 GCAGAAGTAC CTAGTACTTT CTGGGTTGTT TTAGGAGGAG CTTTCTTAGT
     A  E  V  P   S  T  F   W  V  V   L  G  G  A   F  L  V
2251 AACGAGTGGA ACGATTTATA TAAGGAAAAC TAGAAAATGA
       T  S  G   T  I  Y  I   R  K  T   R  K  *
```

EF2713

```
   1 TACAATAGGT AACGAAAGGA ATGATTAAAA TGAAAAAAAT GTTTTTAGGA
                                   M   K  K  M   F  L  G
  51 CTCTTTTGTT TTGTTTCGAT TACCACACTA TCAGTTTCTA ATGTATGGGC
     L  F  C  F   V  S  I   T  T  L   S  V  S  N   V  W  A
 101 AGAAGATTCG GCTAATGAAG CTTCTGTGGA AAAGTCAACA GTTACTTCTT
       E  D  S   A  N  E  A   S  V  E   K  S  T   V  T  S  L
 151 TAACTCAAGA AACTTCAGCA ACAACAATTA ATGCTTCAAC TGATTCAACA
       T  Q  E   T  S  A   T  T  I  N   A  S  T   D  S  T
 201 GCACTTACAG CTGAAAGCGA GGAACTTCCA TCGCTTCGTC AAACACTTCT
     A  L  T  A   E  S  E   E  L  P   S  L  R  Q   T  L  L
 251 TAATTACGTA GGTATGTATG GTTTAACAGA AACCTTGATA AATCGTTTAT
       N  Y  V   G  M  Y  G   L  T  E   T  L  I   N  R  L  S
 301 CTGATGACGA ACTGGACTAC GCAAAAAAAG TTTCGTTTCA TTTTGTAAAC
       D  D  E   L  D  Y   A  K  K  V   S  F  H   F  V  N
 351 CAAGACATCA GTGGAACAGC GAGAATGATT ACTAAATTAT ATGGTGAGAA
     Q  D  I  S   G  T  A   R  M  I   T  K  L  Y   G  E  K
 401 ACCTATCCCG GAGGACTCCT ACTCTACAGA TTACTCAACA TTAACGATTG
       P  I  P   E  D  S  Y   S  T  D   Y  S  T   L  T  I  D
 451 ATGACTTAAA AAATTATTTA CCTCAAATTC GACTTTCGTT AATTTATGTA
       D  L  K   N  Y  L   P  Q  I  R   L  S  L   I  Y  V
 501 TATGATTTAA ATAGTGATGT TGTAAATAAT TTAAGCGATC AAACACTCGT
     Y  D  L  N   S  D  V   V  N  N   L  S  D  Q   T  L  V
 551 CGATTTAATT AATCAAGTCA AGGTAGACTA CGCTAACCAA AATTACCCAT
       D  L  I   N  Q  V  K   V  D  Y   A  N  Q   N  Y  P  S
 601 CTGATGTCCG TGGTGATTAT GGTTTAGCAG CAATGGCAGA TAAAATAAAA
       D  V  R   G  D  Y   G  L  A  A   M  A  D   K  I  K
 651 GCAAACGATT ATACGTCAAT TAATCAAAGT GCTGAAAGTG TTTCTTCGGA
     A  N  D  Y   T  S  I   N  Q  S   A  E  S  V   S  S  D
 701 TACTACTAAT ACAGAATCTA CATTACAAAC AACTACGAGC AGTTCGAAGA
       T  T  N   T  E  S  T   L  Q  T   T  T  S   S  S  K  K
 751 AAGCTACTAC ATCAAGTTCA ACCGAGCACA AAAAGGGGAT ATTTCCCAGT
       A  T  T   S  S  S   T  E  H  K   K  G  I   F  P  S
 801 ACTGGCGAAA AAAAATCAGT TTTATTTACT ATCATTGGAA TCATCTTACT
     T  G  E  K   K  S  V   L  F  T   I  I  G  I   I  L  L
 851 ATCTTTAGTT AGTATATTCA TTATAAAAAA TAAAAAGAAA TAG
       S  L  V   S  I  F  I   I  K  N   K  K  K   *
```

Figure S2. Nucleotide and amino acid sequences of EF1033 and EF2713 after adjustment of the start codon. Note that the middle part (representing 600 residues) of EF1033 is not shown, as indicated by the dotted horizontal line. The N-terminal "extensions" of the proteins due to adjustment of the start codon are printed in bold face. Both new start codons are preceded by putative ribosome binding sites, which are underlined. Predicted signal peptidase I cleavage sites are indicated by vertical arrows. The putative LPxTG motifs are printed bold face and underlined. In both proteins the LPxTG motif is followed by a hydrophobic (italic) region (identified with the TMHMM Server) and positively charged residues at the C-terminus (double underlined), which is characteristic for functional C-terminal anchors that are attached to the cell wall by sortase.
