# Supplementary material for: Identification of surface proteins in Enterococcus faecalis V583
Source: BMC Genomics. 2011 Mar 1;12:135. doi: 10.1186/1471-2164-12-135 (PMC3059304; doi:10.1186/1471-2164-12-135)
Supplement: Additional file 4 — Table S5: Proteome data of the proteins identified by LC-MS analysis after different treatments. [file 1471-2164-12-135-S4.PDF]

Table S5. Proteome data of the proteins identified by LC-MS analysis after the different treatments (untreated, trypsin and trypsin beads for one or two hours) from Enterococcus faecalis V583.

| Sample | Gene | Gene product | Accession nr | Cover -age % | No of unique peptides | Peptide sequence | Best proba- bility score | Best Xcorr score | No of +2H spectra | No of +3H spectra | No of +4H spectra | Δ M ppm | Modification |
|---|---|---|---|---|---|---|---|---|---|---|---|---|---|
| Untreated 1h | EF2556 | fumarate reductase flavoprotein subunit | gi29377044 | 69.70 | 40 | AIDFYDQK | 38.82 | 2.43 | 1 | | | -0.70 | |
| | | | | | | AIDFYDQKGFVEK | 77.33 | 3.24 | 2 | | | 0.73 | |
| | | | | | | AIDFYDQKGFVEKGETIEELAEK | 300.00 | 5.10 | | 3 | 1 | 1.45 | |
| | | | | | | AKAIDFYDQK | 34.02 | 2.34 | 1 | | | -0.03 | |
| | | | | | | AKAVVVTTGGFGANEK | 87.37 | 2.92 | 2 | 3 | | -0.07 | |
| | | | | | | AKAVVVTTGGFGANEKLITQYKPELK | 127.27 | 4.52 | | 2 | 2 | 0.82 | |
| | | | | | | ATIDTWNQDVNAK | 88.20 | 3.91 | 3 | | | 0.93 | |
| | | | | | | AVVVTTGGFANEK | 84.41 | 3.91 | 2 | | | -0.58 | |
| | | | | | | AVVVTTGGFGANEKLITQYKPELK | 97.62 | 3.58 | | 1 | 2 | 0.34 | |
| | | | | | | DDKQFGRTTGMEADLSTAPYYAIK | 24.42 | 2.70 | | 1 | | -1.71 | |
| | | | | | | DKVSAAINALPEK | 58.45 | 3.54 | 2 | | | -0.25 | |
| | | | | | | DKVSAAINALPEKSAYLVFDQGVR | 156.54 | 6.27 | | 1 | 4 | 0.05 | |
| | | | | | | EAGMNPVILEK | 49.31 | 2.34 | 2 | | | 0.08 | |
| | | | | | | EDGTPIKGLYAAGELTGGLHGQNR | 70.07 | 5.07 | 1 | 4 | 3 | 0.79 | |
| | | | | | | EEKIPLFVDADVTDLVEENGQIDGVK | 92.36 | 6.33 | | 4 | 1 | 2.14 | |
| | | | | | | EEKIPLFVDADVTDLVEENGQIDGVKVK | 124.18 | 3.77 | | 2 | 2 | -0.25 | |
| | | | | | | EIQIHPTVQQSDAFLIGEAVR | 86.11 | 3.51 | 2 | 3 | | 0.57 | |
| | | | | | | EIQIHPTVQQSDAFLIGEAVRGEGAILASQKGER | 1.19 | 3.44 | | | 2 | -0.54 | |
| | | | | | | FVNELDTR | 56.29 | 2.66 | 2 | | | 0.02 | |
| | | | | | | GITLSNLTITGGMSEK | 137.27 | 4.26 | 3 | | | 0.22 | |
| | | | | | | GITLSNLTITGGMSEKR | 103.26 | 2.74 | 1 | | | 2.25 | |
| | | | | | | GLYAAGELTGGLHGQNR | 111.26 | 3.96 | 3 | 2 | | -0.54 | |
| | | | | | | IGGNAIADIIIYGR | 131.29 | 4.77 | 172 | 42 | | 0.17 | |
| | | | | | | IGGNAIADIIIYGRQAGTQSAEFASAQK | 23.44 | 3.20 | | 1 | | -3.29 | |

| | | | | | | | | | | | | |
|---|---|---|---|---|---|---|---|---|---|---|---|---|
| | | | | | IGMPADTLKATIDTWNQDVNAK | 131.13 | 5.92 | 4 | 5 | | 1.02 | |
| | | | | | IPLFVDADVTDLVEENGQIDGVKVK | 300.00 | 5.89 | | 3 | 1 | 4.45 | |
| | | | | | LITQYKPELK | 17.59 | 2.26 | 1 | | | 0.20 | |
| | | | | | LITQYKPELKNYVTTNQEGTTGDGIQMIQK | 98.23 | 5.39 | | 3 | 5 | 3.08 | |
| | | | | | MPVAGGNTIK | 67.47 | 2.50 | 2 | | | -0.57 | |
| | | | | | MPVAGGNTIKSSSGMNASQTKFQEK | 48.78 | 3.46 | | 2 | | 5.42 | |
| | | | | | NYVTTNQEGTTGDGIQMIQK | 116.73 | 5.52 | 6 | 11 | | -0.80 | |
| | | | | | QAGTQSAEFASAQK | 13.06 | 2.06 | 1 | | | 0.30 | |
| | | | | | RTHRPADGSAIGGYLVDGLVR | 93.50 | 4.84 | | 5 | 2 | 0.50 | |
| | | | | | SAYLVFDQGVR | 78.38 | 3.29 | 2 | | | 0.48 | |
| | | | | | SAYLVFDQGVRDR | 23.14 | 2.13 | 1 | | | -0.15 | |
| | | | | | TEVLREDGTPIKGLYAAGELTGGLHGQNR | 137.14 | 6.64 | | 2 | 4 | 0.02 | |
| | | | | | THRPADGSAIGGYLVDGLVR | 95.76 | 5.02 | 1 | 4 | 5 | 0.16 | |
| | | | | | TTGMEADLSTAPYYAIK | 105.87 | 3.88 | 6 | | | 0.03 | |
| | | | | | VGGALVDMK | 70.41 | 2.97 | 2 | | | -0.10 | |
| | | | | | VSAAINALPEKSAYLVFDQGVR | 63.41 | 3.78 | | 1 | | 3.09 | |
| EF0123 | hypothetical protein EF0123 | gi29374774 | 14.81 | 5 | GSYGYNVNPVSVATR | 97.82 | 3.46 | 3 | | | 1.23 | |
| | | | | | RPDEIKPNVNYQTHVQNIGWQGVVK | 97.11 | 4.64 | | 1 | 3 | -0.64 | |
| | | | | | THVQEIGWQGYVK | 51.15 | 2.59 | | 1 | | -1.38 | |
| | | | | | VPDINYQTHIQDIGWQGVVK | 116.14 | 4.23 | 1 | 4 | | 0.78 | |
| | | | | | YFGASATDLVITAQSYGR | 144.11 | 6.05 | 2 | | | -0.08 | |
| EF1818 | coccolysin | gi29376362 | 30.59 | 8 | DLVFLAIDKRVnNEGqLFYK | 15.29 | 2.39 | | | 1 | 22.31 | N12(Deamidated) Q16(Deamidated) |
| | | | | | GMPILSVVDEQHPDAYDNAFWDGK | 69.17 | 2.60 | | 1 | | 1.38 | |
| | | | | | IqSVDAIGEEGVK | 6.13 | 2.04 | 1 | | | 4.75 | Q2(Deamidated) |
| | | | | | NSFQVAFNVPVEK | 38.54 | 3.21 | 2 | | | -0.03 | |
| | | | | | QTEGVTVDSDNVIIHLDR | 50.57 | 2.81 | | 1 | | 1.00 | |
| | | | | | TGIRNLQTPSKHGQPETMAQYDDR | 23.86 | 3.59 | | | 2 | 2.36 | |

| | | | | | | | | | | | | |
|---|---|---|---|---|---|---|---|---|---|---|---|---|
| | | | | | VQYGDEAASV VSAAFNSAGIG AK | 47.39 | 5.17 | 2 | | | 3.93 | |
| | | | | | YKGTPYYDQG GVHYNSGIINR | 67.45 | 3.58 | | 1 | 1 | -0.92 | |
| EF2864 | hypothetical protein EF2864 | gi29377332 | 11.03 | 2 | LLITQDSGNYP AEEYYYR | 139.96 | 4.23 | 3 | 3 | | -0.03 | |
| | | | | | WGPTGAALLL YK | 74.72 | 3.37 | 2 | | | 0.34 | |
| EF3041 | pheromone binding protein | gi29377499 | 5.26 | 2 | TLKGDFQIAVR | 23.46 | 2.68 | | 1 | | 0.78 | |
| | | | | | WSDGKPVTAA DYVYGWQR | 85.32 | 3.73 | 2 | 4 | | 0.74 | |
| EF2860 | YkuD putative, pewptidoglycan binding protein | gi29377328 | 10.55 | 2 | FKNNGSYGWSI DGAK | 31.59 | 2.68 | | 1 | | 0.77 | |
| | | | | | IFDVSYDGmPV IIYGHIYDDAPG EFDKPVDYGEE V | 149.55 | 5.45 | | 4 | | 4.98 | M9(Oxidation) |
| EF0394 | secreted antigen, putative | gi29375030 | 6.46 | 2 | ASLALEQSSAE SSK | 103.09 | 3.68 | 2 | | | 1.93 | |
| | | | | | VGFGYSGSTIV GHSA | 63.14 | 3.14 | 3 | | | 1.26 | |
| EF0226 | 50S ribosomal protein L15 | gi29374870 | 8.22 | 1 | LGFEGGQTPLF R | 77.39 | 3.05 | 4 | | | 0.13 | |
| EF0417 | hypothetical protein EF0417 | gi29375051 | 12.50 | 3 | EGEQAYVLVN DFGTIIR | 38.98 | 3.18 | | 1 | | 1.92 | |
| | | | | | NGYHMQATID LGDLGAIELPK K | 1.52 | 3.04 | | | 1 | -0.53 | |
| | | | | | RANIYNKWNR | 76.70 | 3.27 | | 2 | | -0.48 | |
| EF0201 | elongation factor Tu | gi29374847 | 4.05 | 1 | LLDYAEAGDNI GALLR | 121.69 | 4.42 | 4 | | | 1.15 | |
| EF1264 | sulfatase domain-containing protein | gi29375833 | 7.55 | 2 | FIAVSNHYPYS QFTNDEAGFPI AK | 111.99 | 3.75 | | 2 | | 1.78 | |
| | | | | | FYTNSGLKPVN PEDYDYKNQL QQLEAIEK | 40.69 | 2.89 | | | 1 | 2.24 | |
| EF2746 | dltD protein | gi29377221 | 2.83 | 1 | YVPFFGSSELS R | 52.21 | 2.27 | 3 | | | 0.93 | |
| EF0916 | 50S ribosomal protein L20 | gi29375500 | 10.08 | 1 | EQVMNSYYYA FR | 93.01 | 2.90 | 2 | | | 0.97 | |
| EF0164 | putative lipoprotein | gi29374814 | 14.52 | 2 | IISHVGDLYDE K | 54.38 | 2.94 | | 1 | | -1.14 | |
| | | | | | NKIISHVGDLY DEKYQEK | 64.90 | 3.13 | | | 1 | -4.27 | |
| EF2925 | cold-shock domain-contain protein | gi29377389 | 19.70 | 1 | WFNAEKGFGFI SR | 37.57 | 2.77 | 2 | | | 1.34 | |
| EF2144 | putative lipoprotein | gi29376653 | 8.04 | 1 | EANVEIHITPQS ADSGLVEIIDY | 116.55 | 3.65 | 2 | | | 4.23 | |
| EF1046 | pyruvate kinase | gi29375625 | 4.79 | 2 | AVVAATAEEA VAK | 69.20 | 3.46 | 1 | | | 1.06 | |
| | | | | | LVQGQGVGEE AIIAK | 76.59 | 3.29 | 1 | | | 1.54 | |
| EF0200 | elongation factor G | gi29374846 | 2.45 | 1 | VYSGVLESGSY VLNASK | 125.70 | 3.98 | 1 | | | 2.79 | |
| EF0211 | 50S ribosomal protein L22 | gi29374856 | 9,57 | 1 | TSHITVVVTEK | 40.34 | 2.20 | | 1 | | -0.26 | |

| | | | | | | | | | | | | | |
|---|---|---|---|---|---|---|---|---|---|---|---|---|---|
| | EF1167 | fructose-bisphosphate aldolase | gi29375743 | 9.34 | 1 | KGGYAVGGYNTNNLEWTQAILEAAEAK | 71.77 | 5.02 | | 1 | | 3.27 | |
| | EF1308 | dnak protein | gi29375876 | 4.6 | 1 | ePNKSVNPDEVVAmGAAIqGGVITGDVK | 1 | 2.55 | | 1 | | 8.87 | N-Term(Acetyl) M14(Oxidation) Q19(Deamidated) |
| | EF2398 | 30S ribosomal protein S2 | gi29376895 | 3.83 | 1 | FLGGIADMPR | 21.89 | 2.05 | 1 | | | 0.6 | |
| Untreated 2h | EF2556 | fumarate reductase flavoprotein subunit | gi29377044 | 58.81 | 39 | AIDFYDQK | 29.30 | 2.03 | 1 | | | 0.89 | |
| | | | | | | AIDFYDQKGFVEK | 87.86 | 4.09 | 4 | | | 1.83 | |
| | | | | | | AIDFYDQKGFVEKGETIEELAEK | 144.11 | 4.94 | | 2 | 1 | 4.08 | |
| | | | | | | AKAIDFYDQK | 29.65 | 2.78 | 2 | 2 | | -0.47 | |
| | | | | | | AKAIDFYDQKGFVEK | 88.13 | 3.49 | 1 | 2 | | 0.15 | |
| | | | | | | AKAVVVTTGGFGANEK | 61.40 | 3.80 | 2 | 2 | | 0.11 | |
| | | | | | | AKAVVVTTGGFGANEKLITQYKPELK | 63.38 | 2.93 | | | 1 | 3.26 | |
| | | | | | | ATIDTWNQDVNAKDDKQFGR | 72.20 | 3.59 | | 1 | | 3.94 | |
| | | | | | | AVVVTTGGFGANEK | 98.28 | 4.24 | 5 | | | -0.42 | |
| | | | | | | AVVVTTGGFGANEKLITQYKPELK | 50.42 | 4.39 | | 1 | | 9.02 | |
| | | | | | | DKVSAAINALPEK | 91.57 | 4.08 | 3 | | | 0.48 | |
| | | | | | | EAGmNPVILEK | 41.97 | 2.43 | 3 | | | 0.16 | M4(Oxidation) |
| | | | | | | EAGmNPVILEKmPVAGGNTIK | 43.61 | 3.53 | | 2 | | 3.29 | M4(Oxidation) M12(Oxidation) |
| | | | | | | EDGTPIKGLYAAGELTGGLHGQNR | 49.23 | 3.80 | | 1 | 2 | 1.13 | |
| | | | | | | EDGTPIKGLYAAGELTGGLHGQNRIGGNAIADIIIYGR | 144.36 | 4.51 | | | 2 | 3.71 | |
| | | | | | | EEKIPLFVDADVTDLVEENGQIDGVK | 97.46 | 7.15 | | 3 | | 3.24 | |
| | | | | | | EEKIPLFVDADVTDLVEENGQIDGVKVK | 73.29 | 3.74 | | 3 | 3 | -0.01 | |
| | | | | | | GLYAAGELTGGLHGQNR | 123.87 | 5.02 | 3 | 1 | | 1.11 | |
| | | | | | | GLYAAGELTGGLHGQNRIGGNAIADIIIYGR | 77.32 | 3.86 | | | 1 | 3.13 | |
| | | | | | | IGGNAIADIIIYGR | 120.52 | 4.54 | 172 | 17 | | -0.15 | |
| | | | | | | IGGNAIADIIIYGRQAGTQSAEFASAQK | 300.00 | 6.36 | 1 | 4 | | 3.66 | |
| | | | | | | IGmPADTLKATIDTWNQDVNAK | 95.11 | 4.73 | | 3 | | 2.66 | M3(Oxidation) |
| | | | | | | IPLFVDADVTDLVEENGQIDGVK | 85.00 | 5.85 | 1 | | | 7.03 | |
| | | | | | | IPLFVDADVTDLVEENGQIDGVKVK | 150.51 | 6.08 | | 4 | | 3.72 | |

| | | | | | | | | | | | | |
|---|---|---|---|---|---|---|---|---|---|---|---|---|
| | | | | | LITQYKPELK | 40.64 | 2.83 | 2 | | | 0.81 | |
| | | | | | LITQYKPELKNYVTTNQEGTTGDGIQmIQK | 111.11 | 4.38 | | 1 | 7 | 2.55 | M27(Oxidation) |
| | | | | | MPVAGGNTIK | 80.73 | 2.35 | 4 | | | 0.10 | |
| | | | | | MPVAGGNTIKSSGmNASQTKFQEK | 31.17 | 2.87 | | 5 | | 4.40 | M15(Oxidation) |
| | | | | | NYVTTNQEGTTGDGIQmIQK | 114.31 | 5.15 | 5 | 6 | | 1.66 | M17(Oxidation) |
| | | | | | QAGTQSAEFASAQK | 66.85 | 3.10 | 4 | | | 1.40 | |
| | | | | | RTHRPADGSAIGGYLVDGLVR | 132.73 | 4.57 | | 5 | 2 | -0.05 | |
| | | | | | SAYLVFDQGVR | 76.11 | 3.16 | 4 | | | 0.97 | |
| | | | | | SAYLVFDQGVRDR | 10.15 | 2.23 | 1 | | | 1.44 | |
| | | | | | TEVLREDGTPIKGLYAAGELTGGLHGQNR | 300.00 | 7.67 | 2 | 2 | | 1.74 | |
| | | | | | THRPADGSAIGGYLVDGLVR | 103.39 | 5.39 | 2 | 4 | | 0.18 | |
| | | | | | THRPADGSAIGGYLVDGLVRNVR | 43.41 | 2.17 | | | 1 | -0.15 | |
| | | | | | TTGmEADLSTAPYYAIK | 84.68 | 4.09 | 1 | | | 3.29 | M4(Oxidation) |
| | | | | | VGGALVDmK | 59.43 | 2.82 | 2 | | | -0.63 | M8(Oxidation) |
| | | | | | VSAAINALPEKSAYLVFDQGVR | 110.62 | 4.43 | | 1 | | 3.64 | |
| EF2860 | YkuD putative, pewptidoglycan binding protein | gi29377328 | 19.62 | 6 | FKNNGSYGWSIDGAK | 87.92 | 2.16 | 3 | 2 | | 0.47 | |
| | | | | | IANNYIEIDLKDQK | 47.33 | 2.63 | | 1 | | -0.64 | |
| | | | | | IFDVSYDGMPVIIYGHIYDDAPGEFDKPVDYGEEV | 300.00 | 5.67 | | 7 | 2 | 3.44 | |
| | | | | | RFKNNGSYGWSIDGAK | 72.84 | 3.89 | | 4 | 1 | 0.17 | |
| | | | | | VKLPLNEAFKK | 18.42 | 2.09 | | 1 | | -0.16 | |
| | | | | | YNKGTATVPGFHTILYR | 94.39 | 4.29 | | 2 | | 1.98 | |
| EF2864 | hypothetical protein EF2864 | gi29377332 | 11.03 | 2 | LLITQDSGNYPAEEYYYR | 142.39 | 5.07 | 4 | 3 | | 4.80 | |
| | | | | | WGPTGAALLLYK | 71.24 | 3.59 | 2 | | | 0.71 | |
| EF1818 | coccolysin | gi29376362 | 10.59 | 3 | GmPILSVVDEQHPDAYDNAFWDGK | 79.02 | 3.16 | | 2 | | 6.40 | M2(Oxidation) |
| | | | | | TYYEDHFQR | 48.10 | 2.24 | 1 | | | -2.55 | |
| | | | | | YKGTPYYDQGGVHYNSGIINR | 82.59 | 4.63 | | 2 | 3 | 1.59 | |
| EF3041 | pheromone binding protein | gi29377499 | 6.35 | 3 | LREESKWSDGKPVTAADYVYGWQR | 66.67 | 2.87 | | | 2 | 2.51 | |

| | | | | | | | | | | | | | |
|---|---|---|---|---|---|---|---|---|---|---|---|---|---|
| | | | | | | TLKGDFQIAVR | 32.15 | 2.99 | | 2 | | -0.04 | |
| | | | | | | WSDGKPVTAADYVYGWQR | 92.16 | 3.18 | 2 | | | 1.37 | |
| | EF0417 | hypothetical protein EF0417 | gi29375051 | 8.16 | 2 | AIQKEGEQAYVLVNDFGTIIRR | 41.98 | 2.39 | | 1 | | 3.68 | |
| | | | | | | RANIYNKWNR | 64.35 | 3.37 | 1 | 4 | | -0.39 | |
| | EF0164 | putative lipoprotein | gi29374814 | 25.00 | 3 | IISHVGDLYDEKYQEK | 41.58 | 2.25 | | | 1 | -0.40 | |
| | | | | | | SHGNYEVIYK | 44.35 | 2.30 | 1 | | | 1.60 | |
| | | | | | | SHGNYEVIYKSGKFK | 22.09 | 2.64 | | 1 | 2 | -0.23 | |
| | EF2144 | putative lipoprotein | gi29376653 | 12.59 | 2 | EANVEIHITPQSADSGLVEIIDY | 128.03 | 4.66 | 4 | | | 3.99 | |
| | | | | | | HQFNDmYPYKGSK | 19.55 | 2.88 | | 1 | | -0.66 | M6(Oxidation) |
| | EF0123 | hypothetical protein EF0123 | gi29374774 | 5.75 | 2 | RPDEIKPNVNYQTHVQNIGWQGVVK | 109.73 | 4.03 | | | 2 | 2.29 | |
| | | | | | | VPDINYQTHIQDIGWQGVVK | 87.05 | 3.78 | | 2 | | -1.78 | |
| | EF1167 | fructose-bisphosphate aldolase | gi29375743 | 9.69 | 2 | GGYAVGGYNTNNLEWTQAILEAAEAKK | 1.00 | 2.27 | | 1 | | 3.82 | |
| | | | | | | KGGYAVGGYNTNNLEWTQAILEAAEAK | 119.91 | 6.13 | | 2 | | 3.82 | |
| | EF2925 | cold-shock domain-contain protein | gi29377389 | 30.30 | 2 | mEqGTVKWFnAEKGFGFISR | 1.00 | 3.29 | | 1 | | 14.54 | N-Term(Acetyl) Q3(Deamidated) N10(Deamidated) |
| | | | | | | WFNAEKGFGFISR | 70.24 | 3.72 | 2 | | | 2.81 | |
| | EF0226 | 50S ribosomal protein L15 | gi29374870 | 12.33 | | LGFEGGQTPLFR | 64.05 | 2.59 | 1 | | | 1.47 | |
| | | | | | | SGGGVRLGFEGGQTPLFR | 75.74 | 3.76 | | 1 | | -0.60 | |
| | EF0201 | elongation factor Tu | gi29374847 | 7.59 | 2 | FKAEVYVLSK | 29.06 | 2.76 | | 1 | | -1.19 | |
| | | | | | | LLDYAEAGDNIGALLRGVAR | 12.52 | 2.17 | | 1 | | 4.45 | |
| | EF2746 | dltD protein | gi29377221 | 6.37 | 2 | AINNNKFEISNGFYR | 37.52 | 2.23 | | 1 | | 0.25 | |
| | | | | | | YVPFFGSSELSR | 18.08 | 2.30 | 1 | | | 0.81 | |
| | EF0211 | 50S ribosomal protein L22 | gi29374856 | 17.39 | 2 | GSASPINKRTSHITVVVTEK | 38.27 | 2.21 | | | 1 | -1.55 | |
| | | | | | | TSHITVVVTEK | 25.47 | 2.65 | 1 | | | 0.28 | |
| | EF0916 | 50S ribosomal protein L20 | gi29375500 | 10.08 | 1 | EQVmNSYYYAFR | 77.98 | 2.84 | 1 | | | 3.37 | M4(Oxidation) |
| Trypsin 1h | EF2556 | fumarate reductase flavoprotein subunit | gi29377044 | 67.72 | 40 | AIDFYDQK | 60.47 | 2.40 | 3 | | | -0.15 | |
| | | | | | | AIDFYDQKGFVEKGETIEELAEK | 125.70 | 4.76 | | 2 | 3 | 1.94 | |
| | | | | | | AKAIDFYDQK | 31.09 | 2.05 | 1 | | | -0.28 | |

| | | | | | | | |
|---|---|---|---|---|---|---|---|
| AKAIDFYDQKGFVEK | 1.00 | 2.45 | | | 1 | -1.26 | |
| AKAVVVTTGGFGANEK | 63.46 | 3.64 | 3 | 3 | | -0.99 | |
| ATIDTWNQDVNAK | 84.42 | 3.14 | 4 | | | 1.23 | |
| ATIDTWNQDVNAKDDKQFGR | 28.01 | 2.32 | | | 1 | -0.53 | |
| AVVVTTGGFGANEK | 78.16 | 3.74 | 8 | 1 | | -0.03 | |
| DDKQFGRTTGMEADLSTAPYYAIK | 95.87 | 3.88 | | 2 | | 1.77 | |
| DKVSAAINALPEK | 96.69 | 3.92 | 2 | | | 0.24 | |
| DKVSAAINALPEKSAYLVFDQGVR | 300.00 | 6.92 | 2 | 3 | 3 | 0.66 | |
| EAGMNPVILEK | 35.82 | 2.29 | 1 | | | 0.94 | |
| EDGTPIKGLYAAGELTGGLHGQNR | 137.14 | 4.97 | 2 | 5 | 3 | 0.72 | |
| EEKIPLFVDADVTDLVEENGQIDGVK | 87.53 | 6.13 | | 4 | 1 | 1.04 | |
| EEKIPLFVDADVTDLVEENGQIDGVKVK | 93.37 | 3.98 | | 2 | 1 | 0.23 | |
| EGIKDSNDKFFEETLK | 40.49 | 2.82 | | 1 | | 2.65 | |
| EIQIHPTVQQSDAFLIGEAVR | 83.00 | 4.42 | 4 | 3 | | 0.02 | |
| EIQIHPTVQQSDAFLIGEAVRGEGAILASQKGER | 3.91 | 2.79 | | | 1 | -14.58 | |
| FVNELDTR | 32.99 | 2.68 | 2 | | | -0.72 | |
| GEGAILASQK | 43.78 | 2.41 | 1 | | | -0.23 | |
| GFVEKGETIEELAEK | 80.34 | 4.30 | 2 | 2 | | -0.23 | |
| GITLSNLTITGGMSEK | 121.92 | 4.04 | 4 | | | 1.03 | |
| GITLSNLTITGGMSEKR | 97.43 | 2.82 | 2 | 2 | | -0.27 | |
| GLYAAGELTGGLHGQNR | 124.90 | 4.52 | 5 | 3 | | -0.03 | |
| IGGNAIADIIIYGR | 121.17 | 4.56 | 188 | 63 | | 0.04 | |
| IGmPADTLKATIDTWNQDVNAK | 300.00 | 4.74 | | 6 | | 1.75 | M3(Oxidation) |
| IPLFVDADVTDLVEENGQIDGVK | 106.12 | 5.12 | 4 | 9 | | 2.99 | |
| IPLFVDADVTDLVEENGQIDGVKVK | 138.68 | 5.29 | | 5 | 2 | -0.75 | |
| LITQYKPELKNYVTTNQEGTTGDGIQmIQK | 52.69 | 3.39 | | | 3 | -0.38 | M27(Oxidation) |
| NYVTTNQEGTTGDGIQMIQK | 140.62 | 4.96 | 8 | 7 | | -0.17 | |

| | | | | | | | | | | | | |
|---|---|---|---|---|---|---|---|---|---|---|---|---|
| | | | | | RTHRPADGSAI GGYLVDGLVR | 121.96 | 4.52 | | 5 | 5 | -0.02 | |
| | | | | | SAYLVFDQGV R | 73.16 | 3.35 | 5 | | | 0.24 | |
| | | | | | SAYLVFDQGV RDR | 24.70 | 2.20 | 1 | | | 0.58 | |
| | | | | | TEVLREDGTPI KGLYAAGELT GGLHGQNR | 300.00 | 6.80 | | 2 | 4 | -0.09 | |
| | | | | | THRPADGSAIG GYLVDGLVR | 107.35 | 5.42 | 3 | 5 | 5 | -0.01 | |
| | | | | | TTGMEADLST APYYAIK | 99.49 | 4.06 | 8 | 2 | | 1.13 | |
| | | | | | TTGMEADLST APYYAIKIAPGI HHTMGGVK | 45.60 | 3.02 | | 1 | 1 | 3.07 | |
| | | | | | VGGALVDmK | 71.69 | 3.10 | 2 | | | 3.10 | M8(Oxidation) |
| | | | | | VSAAINALPEK | 45.52 | 2.73 | 1 | | | -0.47 | |
| | | | | | VSAAINALPEK SAYLVFDQGV R | 71.51 | 4.69 | | 2 | | 1.63 | |
| EF0123 | hypothetical protein EF0123 | gi29374774 | 21.07 | 9 | AFLVGDEAR | 37.11 | 2.23 | 1 | | | -1.60 | |
| | | | | | GSYGYNVNPV SVATR | 97.22 | 3.69 | 4 | | | 1.23 | |
| | | | | | LTGEIANAYDV YYR | 112.42 | 3.42 | 3 | | | 1.55 | |
| | | | | | NGYTLTYDPY GR | 56.05 | 2.64 | 3 | | | 0.89 | |
| | | | | | RPDEIKPNVNY QTHVQNIGWQ GVVK | 125.36 | 5.12 | | 1 | 4 | -0.26 | |
| | | | | | THVQEIGWQG YVK | 55.00 | 2.94 | 2 | 2 | | -0.64 | |
| | | | | | VPDINYQTHIQ DIGWQGVVK | 64.37 | 3.73 | | 2 | | 0.41 | |
| | | | | | YFGASATDLVI TAQSYGR | 141.16 | 4.57 | 4 | 1 | | 2.00 | |
| | | | | | YYDVYYR | 41.92 | 2.38 | 1 | | | 0.45 | |
| EF0201 | elongation factor Tu | gi29374847 | 40.00 | 11 | DLLSEYDFPGD DVPVIAGSALK | 148.41 | 4.52 | 1 | | | 5.11 | |
| | | | | | FKAEVYVLSK | 52.32 | 2.10 | 1 | 2 | | 0.19 | |
| | | | | | GITINTSHIEYE TETR | 101.30 | 4.12 | 1 | 1 | | 1.66 | |
| | | | | | GQVLAKPATIT PHTKFKAEVY VLSK | 14.05 | 2.89 | | | 1 | 1.69 | |
| | | | | | HYAHVDCPGH ADYVK | 37.86 | 2.72 | | | 1 | -1.39 | |
| | | | | | KLLDYAEAGD NIGALLR | 83.99 | 4.47 | 2 | 2 | | 0.90 | |
| | | | | | LLDYAEAGDNI GALLR | 121.69 | 4.97 | 3 | 1 | | 1.39 | |
| | | | | | NmITGAAQmD GAILVVSAADG PmPQTR | 17.58 | 3.08 | | 1 | | 1.90 | M2(Oxidation) M9(Oxidation) M23(Oxidation) |

| | | | | | | | | | | | | |
|---|---|---|---|---|---|---|---|---|---|---|---|---|
| | | | | | TTLTAAIATVLSK | 80.00 | 3.12 | 1 | | | 0.07 | |
| | | | | | TVGSGVVTEIVK | 20.36 | 2.30 | 1 | | | 0.15 | |
| | | | | | VGDEVEIVGIK | 56.84 | 2.36 | 1 | | | -0.87 | |
| EF1818 | coccolysin | gi29376362 | 26.47 | 8 | GmPILSVVDEQHPDAYDNAFWDGK | 127.13 | 3.26 | | 3 | | 2.84 | M2(Oxidation) |
| | | | | | IGYTIIQNLGIEK | 90.74 | 4.22 | 2 | | | 0.50 | |
| | | | | | IqSVDAIGEEGVK | 10.76 | 2.00 | 1 | | | 4.02 | Q2(Deamidated) |
| | | | | | NSFQVAFNVPVEK | 37.13 | 2.95 | 1 | | | 1.44 | |
| | | | | | QTEGVTVDSDNVIIHLDR | 56.01 | 3.26 | | 1 | | 0.82 | |
| | | | | | VNNEGQLFYK | 41.97 | 2.23 | 1 | | | 1.19 | |
| | | | | | VQYGDEAASVVSAAFNSAGIGAK | 113.17 | 5.30 | 3 | | | 3.44 | |
| | | | | | YKGTPYYDQGGVHYNSGIINR | 63.60 | 3.76 | | 2 | 3 | 0.06 | |
| EF1264 | sulfatase domain-containing protein | gi29375833 | 21.37 | 10 | ADWNDFDNANmQR | 99.94 | 3.05 | 1 | | | 2.69 | M11(Oxidation) |
| | | | | | FIAVSNHYPYSQFTNDEAGFPIAK | 121.35 | 3.90 | 1 | 2 | | 4.53 | |
| | | | | | FYTNSGLKPVNPEDYDYK | 80.12 | 3.50 | | 1 | | 1.82 | |
| | | | | | FYTNSGLKPVNPEDYDYKNQLQQLEAIEK | 39.58 | 3.92 | | | 1 | 6.39 | |
| | | | | | nqLqqLEAIEKEK | 1.00 | 2.14 | 1 | | | 13.80 | N-Term(Acetyl) Q2(Deamidated) Q4(Deamidated) Q5(Deamidated) |
| | | | | | NYIQLGQDLFSK | 59.75 | 3.20 | 2 | | | 0.17 | |
| | | | | | QLETSDQITNGDLLR | 103.92 | 2.93 | 2 | | | 2.52 | |
| | | | | | STFSFDNFFHQVGQGK | 93.34 | 3.96 | 2 | | | 3.52 | |
| | | | | | TSDAETLLENSLFGLDQGSLFTQVGGK | 72.87 | 4.80 | | 1 | | 7.58 | |
| | | | | | YTILGSSIYDTK | 33.85 | 3.05 | 1 | | | 0.50 | |
| EF2860 | YkuD putative, pewptidoglycan binding protein | gi29377328 | 20.04 | 5 | FKNNGSYGWSIDGAK | 17.96 | 2.91 | | 1 | | -0.33 | |
| | | | | | IFDVSYDGMPVIIYGHIYDDAPGEFDKPVDYGEEV | 300.00 | 5.06 | | 4 | | 3.15 | |
| | | | | | RFKNNGSYGWSIDGAK | 25.00 | 2.69 | | 1 | | -0.56 | |
| | | | | | TQELLVnALnSqEqTNAITAPLVGDTK | 1.00 | 2.58 | | | 4 | 18.27 | |
| | | | | | YNKGTATVPGFHTILYR | 73.92 | 4.18 | | 2 | 1 | -0.03 | |
| EF2864 | hypothetical protein EF2864 | gi29377332 | 20.22 | 3 | LLITQDSGNYPAEEYYYR | 137.27 | 4.59 | 4 | 4 | | 0.70 | |

| | | | | | | | | | | | |
|---|---|---|---|---|---|---|---|---|---|---|---|
| | | | | | TLTDAENNDTNLGFLGmNGNDFFFR | 46.45 | 3.87 | | 1 | 4.13 | M17(Oxidation) |
| | | | | | WGPTGAALLLYK | 86.58 | 3.60 | 2 | | -0.27 | |
| EF0200 | elongation factor G | gi29374846 | 16.88 | 7 | GQYGHVWVEFTPNEEGK | 11.31 | 2.64 | | 1 | -2.26 | |
| | | | | | IGADFFYSVESLHDR | 50.47 | 3.00 | | 1 | -1.55 | |
| | | | | | IGETHEGASQMDWMEQEQER | 50.23 | 4.02 | | 2 | -2.14 | |
| | | | | | LYDGSYHDVDSNETAFR | 35.19 | 2.58 | | 1 | 2.89 | |
| | | | | | VNIIDTPGHVDFTIEVQR | 114.69 | 3.53 | | 2 | -0.96 | |
| | | | | | VYSGDIAAAVGLK | 35.59 | 2.50 | 1 | | 1.95 | |
| | | | | | VYSGVLESGSYVLNASK | 101.96 | 4.18 | 1 | | 3.52 | |
| EF3041 | pheromone binding protein | gi29377499 | 5.26 | 2 | TLKGDFQIAVR | 39.99 | 2.95 | | 3 | -0.41 | |
| | | | | | WSDGKPVTAADYVYGWQR | 117.87 | 3.88 | 2 | 1 | 0.88 | |
| EF0417 | hypothetical protein EF0417 | gi29375051 | 15.31 | 4 | EGEQAYVLVNDFGTIIR | 92.53 | 3.89 | 1 | 1 | -1.56 | |
| | | | | | MAIESGLESADR | 39.17 | 2.46 | 1 | | 1.48 | |
| | | | | | NGYHMQATIDLGDLGAIELPK | 19.53 | 3.04 | | 1 | -2.47 | |
| | | | | | RANIYNKWNR | 51.12 | 3.17 | | 2 | -0.67 | |
| EF1964 | glyceraldehyde-3-phosphate dehydrogenase | gi29376486 | 15.92 | 3 | IQDVEGIEVVAINDLTDAK | 300.00 | 5.87 | 2 | | 2.37 | |
| | | | | | TVAWYDNEmSYTAQLVR | 114.74 | 3.98 | 2 | | 5.10 | M9(Oxidation) |
| | | | | | VPVATGSLTELVTVLDK | 145.40 | 5.33 | 2 | | 1.34 | |
| EF0177 | hypothetical protein EF0177 | gi29374827 | 21.05 | 5 | ALAAAmYQNGVDIIFHASGATGQGVFQEAK | 97.36 | 6.35 | | 2 | 1.21 | M6(Oxidation) |
| | | | | | FNTIFGIGYLLK | 43.30 | 2.56 | 1 | | 1.79 | |
| | | | | | GVGTAVQDIANR | 37.08 | 2.28 | 1 | | 3.09 | |
| | | | | | VGFVGGEEGVVIDR | 55.06 | 2.86 | 1 | | 0.66 | |
| | | | | | VWVIGVDR | 49.32 | 2.07 | 1 | | -0.40 | |
| EF0228 | adenylate kinase | gi29374872 | 15.28 | 2 | IIDTYGIPHISTGDMFR | 46.21 | 3.14 | | 2 | -1.02 | |
| | | | | | LAVNIESSAPILAFYK | 124.95 | 4.52 | 3 | | 1.19 | |
| EF1961 | enolase | gi29376483 | 8.80 | 2 | AAADYLEVPLYHYLGGFNTK | 109.75 | 3.80 | 1 | 3 | 0.97 | |
| | | | | | GNPTIEVEVYTESGAFGR | 43.45 | 2.74 | 1 | | 1.52 | |

| | | | | | | | | | | | |
|---|---|---|---|---|---|---|---|---|---|---|---|
| EF0176 | hypothetical protein EF0176 | gi29374826 | 14.85 | 3 | ALASSmYQAGADIIYHAAATTGQGIFQEAK | 109.32 | 5.46 | | 1 | -4.22 | M6(Oxidation) |
| | | | | | SFNQSAWEGMQEWGK | 106.43 | 3.41 | 3 | | 3.42 | |
| | | | | | VWVIGVDR | 49.32 | 2.07 | 1 | | -0.40 | |
| EF2718 | 50S ribosomal protein L1 | gi29377196 | 14.41 | 2 | LVENFNTINDVLLK | 83.34 | 2.48 | 1 | | 0.92 | |
| | | | | | NISVTTTFGPGIHVDQASF | 104.17 | 3.84 | 3 | | 1.49 | |
| EF0205 | 30S ribosomal protein S10 | gi29374850 | 12.75 | 2 | LDLPSGVNIEIK | 91.86 | 2.79 | 3 | | 0.36 | |
| | | | | | LDLPSGVNIEIKL | 34.62 | 2.51 | 1 | | 1.49 | |
| EF0226 | 50S ribosomal protein L15 | gi29374870 | 17.12 | 2 | AGIKVLADGELTK | 16.48 | 2.72 | | 1 | 0.06 | |
| | | | | | LGFEGGQTPLFR | 79.26 | 3.21 | 3 | | 0.98 | |
| EF0164 | putative lipoprotein | gi29374814 | 17.74 | 2 | IISHVGDLYDEK | 40.27 | 2.78 | | 2 | 0.69 | |
| | | | | | SHGNYEVIYK | 28.94 | 2.41 | 1 | 1 | -0.36 | |
| EF_B0004 | TraC protein | gi29377898 | 6.43 | 2 | ELSATTmFLEVNqRnK | 18.45 | 2.51 | | 2 | -18.71 | M7(Oxidation) Q13(Deamidated) N15(Deamidated) |
| | | | | | WADGTDITADDFVTAWQR | 113.41 | 4.79 | 2 | | 2.65 | |
| EF1167 | fructose-bisphosphate aldolase | gi29375743 | 13.84 | 2 | KGGYAVGGYNTNNLEWTQAILEAAEAK | 42.15 | 2.29 | | 1 | 3.27 | |
| | | | | | VNVNTEFQLSFAK | 85.79 | 3.34 | 3 | | -1.07 | |
| EF0970 | 50S ribosomal protein L27 | gi29375553 | 34.74 | 2 | IYPGVNVGIGGDDTLFAK | 83.77 | 2.51 | 2 | | 2.71 | |
| | | | | | SADGQTVTGGSILYR | 22.76 | 2.66 | 1 | | 1.08 | |
| EF0916 | 50S ribosomal protein L20 | gi29375500 | 27.73 | 2 | EQVMNSYYYAFR | 81.48 | 3.31 | 2 | | -0.50 | |
| | | | | | MLADLAVNDAAAFTALAEQAK | 148.41 | 3.90 | 1 | | 3.42 | |
| EF1319 | hypothetical protein EF1319 | gi29375887 | 7.76 | 1 | NLTTLYQNPVNTPYMTK | 86.68 | 4.34 | 3 | | -1.31 | |
| EF2144 | putative lipoprotein | gi29376653 | 8.04 | 1 | EANVEIHITPQSADSGLVEIIDY | 107.61 | 4.13 | 2 | 1 | 3.42 | |
| EF1420 | hypothetical protein EF1420 | gi29375987 | 10.40 | 2 | SMSSNLGTLIK | 28.62 | 2.10 | 1 | | -0.24 | |
| | | | | | TSAEIQLGISKYGIQ | 72.52 | 3.49 | 2 | | 1.19 | |
| EF1613 | formate acetyltransferase | gi29376172 | 4.81 | 3 | DmQFFGAR | 36.20 | 2.42 | 1 | | 0.75 | M2(Oxidation) |
| | | | | | FRDLTPEQQADVISR | 44.50 | 2.32 | | 1 | -1.30 | |
| | | | | | IALYGIDYLMEQK | 100.36 | 2.79 | 1 | | 1.30 | |
| EF1308 | dnak protein | gi29375876 | 7.22 | 2 | ePNKSVNPDEVVAmGAAIqGGVITGDVK | 1.00 | 2.20 | | 1 | 25.32 | N-Term(Acetyl) M14(Oxidation) Q19(Deamidated) |

| | | | | | | | | | | | | |
|---|---|---|---|---|---|---|---|---|---|---|---|---|
| | | | | | SYTPQEVSAmI LQYLK | 98.70 | 3.64 | 1 | | | 2.63 | M10(Oxidation) |
| EF0206 | 50S ribosomal protein L3 | gi29374851 | 16.75 | 2 | VDVFQAGDVV DVTGTTK | 27.65 | 2.05 | 1 | | | 1.52 | |
| | | | | | YHRRPGSMGP VAPNRVFK | 25.26 | 2.70 | | | 1 | -1.40 | |
| EF3256 | pheromone cAD1 precursor lipoprotein | gi29377699 | 3.88 | 1 | NGYRAVFEMT VK | 21.53 | 2.24 | 1 | 1 | | -0.25 | |
| EF0234 | 50S ribosomal protein L17 | gi29374878 | 14.96 | 2 | DITTDLIINER | 59.17 | 2.98 | 1 | | | 0.08 | |
| | | | | | LFNDLGPR | 38.59 | 2.18 | 1 | | | -0.39 | |
| EF2746 | dltD protein | gi29377221 | 4.95 | 2 | FEISNGFYR | 36.37 | 2.09 | 1 | | | -0.18 | |
| | | | | | YVPFFGSSELS R | 70.23 | 2.32 | 1 | | | 1.18 | |
| EF0195 | phosphoglycera te mutase 1 | gi29374841 | 10.53 | 2 | ALPFWQDEIAP ALK | 50.54 | 2.25 | 1 | | | 1.27 | |
| | | | | | YGDEQVHIWR | 51.67 | 2.87 | | 1 | | -0.53 | |
| EF0071 | putative lipoprotein | gi29374726 | 8.47 | 3 | ELLGGFAGPLII AEEYPVNLAAS LNK | 6.40 | 3.10 | | | | 7.41 | |
| | | | | | GKGTEGWLPL WAK | 9.07 | 2.26 | | | | 0.08 | |
| | | | | | TNGVYDTNYF NNFSDLGAWH GYYLPEK | 300.00 | 4.77 | | | | 5.81 | |
| EF1523 | hypothetical protein EF1523 | gi29376088 | 2.03 | 1 | GYVVPGGYSL EPAK | 38.69 | 2.57 | 2 | | | 0.05 | |
| EF0304 | putative lipoprotein | gi29374943 | 8.52 | 1 | TAIYGIQLNVE EVAK | 71.77 | 3.55 | 2 | | | 2.35 | |
| EF0223 | 50S ribosomal protein L18 | gi29374867 | 19.49 | 1 | NIYAQVIDDVA GVTLASASALD K | 92.98 | 5.76 | | 2 | | 1.15 | |
| EF1046 | pyruvate kinase | gi29375625 | 4.79 | 2 | AVVAATAEEA VAK | 69.20 | 2.83 | 1 | | | 1.43 | |
| | | | | | LVQGQGVGEE AIIAK | 85.64 | 3.25 | 1 | | | 1.29 | |
| EF1033 | lipoamidase | gi29375612 | 4.66 | 2 | EGLPLGIQFNS ALNEDR | 34.25 | 2.2 | 1 | | | 1.89 | |
| | | | | | ALQDTGQPFLG VPLLLK | 47.61 | 3.24 | 2 | | | 2.3 | |
| EF2398 | 30S ribosomal protein S2 | gi29376895 | 11.49 | 2 | INAmEEDGTFE VLPK | 38.67 | 3.84 | 1 | | | 2.01 | M4(Oxidation) |
| | | | | | WLGGTLTNWD TIQKR | 55.36 | 2.77 | 1 | | | 4.55 | |
| EF1898 | 50S ribosomal protein L19 | gi29376426 | 23.48 | 1 | mNPLIQELTQE QLRTDIPAFRP GDTVR | 1.00 | 2.54 | | 1 | | 28.83 | N-Term(Acetyl) |
| EF0207 | 50S ribosomal protein L4 | gi29374852 | 5.31 | 1 | GGGVVFGPTPR | 9.66 | 2.07 | 1 | | | 0.20 | |
| EF0991 | penicillin-binding protein C | gi29375573 | 2.56 | 1 | LILITNGTNYmP DLTGWSK | 103.37 | 4.10 | 1 | | | -8.82 | M11(Oxidation) |
| EF0221 | 50S ribosomal protein L6 | gi29374866 | 8.99 | 1 | ANFNNmVVGV SEGFQK | 67.81 | 2.85 | 1 | | | 0.06 | M6(Oxidation) |
| EF0218 | 50S ribosomal protein L5 | gi29374863 | 6.70 | 1 | ELLAQLGmPFQ K | 32.78 | 2.83 | 1 | | | -0.18 | |

| | | | | | | | | | | | | | |
|---|---|---|---|---|---|---|---|---|---|---|---|---|---|
| | | | | | | WADGTDITADDFVTAWQR | 138.54 | 5.00 | 3 | | | 2.40 | 2.40 |
| Trypsin 2h | EF2556 | fumarate reductase flavoprotein subunit | gi29377044 | 72.87 | 41 | AIDFYDQK | 39.00 | 2.76 | 3 | | | 0.04 | |
| | | | | | | AIDFYDQKGFVEK | 80.48 | 3.65 | 1 | | | 2.19 | |
| | | | | | | AIDFYDQKGFVEKGETIEELAEK | 92.12 | 3.78 | | 2 | 2 | 1.16 | |
| | | | | | | AKAIDFYDQK | 37.63 | 2.64 | 1 | 2 | | -0.57 | |
| | | | | | | AKAVVVTTGGFGANEK | 99.95 | 3.56 | 3 | 2 | | -0.07 | |
| | | | | | | ATIDTWNQDVNAK | 110.81 | 3.31 | 4 | | | 0.87 | |
| | | | | | | ATIDTWNQDVNAKDDKQFGR | 30.89 | 2.20 | | 2 | | 0.28 | |
| | | | | | | AVVVTTGGFGANEK | 112.21 | 3.77 | 4 | | | -0.42 | |
| | | | | | | DKVSAAINALPEK | 89.94 | 4.15 | 4 | 2 | | 0.07 | |
| | | | | | | DKVSAAINALPEKSAYLVFDQGVR | 148.41 | 6.36 | 1 | 3 | 1 | 2.02 | |
| | | | | | | DSNDKFFEETLK | 39.12 | 2.76 | 2 | 2 | | -0.09 | |
| | | | | | | EDGTPIKGLYAAGELTGGLHGQNR | 68.21 | 5.40 | 1 | 3 | 3 | 1.63 | |
| | | | | | | EEKIPLFVDADVTDLVEENGQIDGVK | 108.30 | 6.92 | | 4 | | 5.44 | |
| | | | | | | EEKIPLFVDADVTDLVEENGQIDGVKVK | 39.50 | 2.20 | | 1 | | 7.27 | |
| | | | | | | EGIKDSNDKFFEETLK | 53.63 | 2.29 | 1 | | | 2.79 | |
| | | | | | | EIQIHPTVQQSDAFLIGEAVR | 107.72 | 4.38 | 4 | 6 | 1 | 2.21 | |
| | | | | | | FVNELDTR | 52.04 | 2.78 | 4 | | | 0.14 | |
| | | | | | | GEGAILASQK | 34.17 | 2.49 | 2 | | | 0.02 | |
| | | | | | | GFVEKGETIEELAEK | 106.16 | 4.29 | 3 | 1 | | -0.13 | |
| | | | | | | GITLSNLTITGGmSEK | 91.69 | 4.56 | 4 | | | 2.01 | M13(Oxidation) |
| | | | | | | GITLSNLTITGGMSEKR | 98.11 | 2.16 | 1 | | | 5.79 | |
| | | | | | | GLYAAGELTGGLHGQNR | 124.90 | 4.62 | 7 | 5 | | -0.17 | |
| | | | | | | IGGNAIADIIIYGR | 127.37 | 4.68 | 159 | 41 | | 0.03 | |
| | | | | | | IGGNAIADIIIYGRQAGTQSAEFASAQK | 148.41 | 5.10 | | 1 | | 5.86 | |
| | | | | | | IGMPADTLKATIDTWNQDVNAK | 91.78 | 4.02 | | 4 | | 2.58 | |
| | | | | | | IPLFVDADVTDLVEENGQIDGVK | 130.52 | 5.77 | 4 | 15 | | -1.04 | |

| | | | | | | | | | | | | |
|---|---|---|---|---|---|---|---|---|---|---|---|---|
| | | | | | IPLFVDADVTD LVEENGQIDGV KVK | 300.00 | 5.76 | | 2 | | 5.19 | |
| | | | | | LITQYKPELK | 49.79 | 2.72 | 2 | | | -0.28 | |
| | | | | | MPVAGGNTIK | 65.67 | 2.47 | 3 | | | -0.20 | |
| | | | | | NYVTTNQEGT TGDGIQMIQK | 137.27 | 5.62 | 7 | 7 | | 0.93 | |
| | | | | | RTHRPADGSAI GGYLVDGLVR | 102.07 | 4.81 | | 13 | 6 | 0.44 | |
| | | | | | SAYLVFDQGV R | 75.16 | 3.37 | 11 | 1 | | -0.13 | |
| | | | | | SAYLVFDQGV RDR | 12.03 | 2.36 | 1 | | | -5.03 | |
| | | | | | TEVLREDGTPI KGLYAAGELT GGLHGQNR | 127.96 | 5.76 | | 1 | 2 | 2.57 | |
| | | | | | THRPADGSAIG GYLVDGLVR | 120.40 | 5.56 | 3 | 12 | 2 | 0.41 | |
| | | | | | TTGMEADLST APYYAIK | 110.26 | 4.19 | 7 | 1 | | 1.74 | |
| | | | | | TTGMEADLST APYYAIKIAPGI HHTmGGVK | 10.89 | 2.24 | | | 1 | 2.47 | M26(Oxidation) |
| | | | | | VGGALVDmK | 58.20 | 2.55 | 2 | | | -0.09 | M8(Oxidation) |
| | | | | | VSAAINALPEK | 45.58 | 2.17 | | 1 | | 1.92 | |
| | | | | | VSAAINALPEK SAYLVFDQGV R | 109.94 | 3.75 | | | 1 | 4.01 | |
| | | | | | YFVDHSAEAID WLDTK | 106.62 | 3.39 | 3 | | | 2.96 | |
| EF1264 | sulfatase domain-containing protein | gi29375833 | 26.07 | 11 | AVEEFFNYLK | 45.29 | 2.50 | 2 | | | 0.81 | |
| | | | | | FIAVSNHYPYS QFTNDEAGFPI AK | 98.22 | 4.59 | 1 | 4 | | 4.53 | |
| | | | | | FYTNSGLKPVN PEDYDYK | 16.15 | 3.13 | | 2 | | -0.74 | |
| | | | | | NYIQLGQDLFS K | 39.21 | 2.74 | 2 | | | 0.66 | |
| | | | | | QLETSDQITNG DLLR | 65.37 | 2.43 | 1 | | | 4.83 | |
| | | | | | QTQGYTSAAF HGNAGNFWNR | 61.20 | 2.97 | 1 | 2 | | 3.89 | |
| | | | | | QTQGYTSAAF HGNAGNFWNR NETYKR | 16.89 | 2.10 | | | 1 | 2.67 | |
| | | | | | SSGLYENSVIV LYGDHYGVSN SR | 69.88 | 3.85 | | 2 | | 6.25 | |
| | | | | | STFSFDNFFHQ VGQGK | 110.22 | 4.53 | 2 | 1 | | 1.00 | |
| | | | | | TSDAETLLENS LFGLDQGSLFT QVGGK | 56.18 | 4.51 | | 1 | | 0.99 | |
| | | | | | YTILGSSIYDTK | 43.07 | 3.51 | 1 | | | -11.22 | |
| EF0201 | elongation factor Tu | gi29374847 | 29.37 | 8 | DLLSEYDFPGD DVPVIAGSALK | 139.38 | 4.94 | 3 | | | 4.86 | |

| | | | | | | | | | | | | |
|---|---|---|---|---|---|---|---|---|---|---|---|---|
| | | | | | FKAEVYVLSK | 81.09 | 3.30 | 1 | 2 | | -0.55 | |
| | | | | | GITINTSHIEYETETR | 33.46 | 2.86 | | 1 | | 2.02 | |
| | | | | | KLLDYAEAGDNIGALLR | 97.35 | 4.63 | 4 | 1 | | 1.74 | |
| | | | | | LLDYAEAGDNIGALLR | 128.50 | 4.44 | 5 | | | 1.03 | |
| | | | | | NmITGAAQmDGAILVVSAADGPmPQTR | 53.56 | 3.98 | | 1 | | -20.44 | M2(Oxidation) M9(Oxidation) M23(Oxidation) |
| | | | | | NVGVPYIVVFLNK | 61.78 | 2.59 | 2 | | | 2.45 | |
| | | | | | VGDEVEIVGIK | 74.12 | 2.62 | 3 | | | 0.35 | |
| EF2860 | YkuD putative, pewptidoglycan binding protein | gi29377328 | 23.21 | 9 | FKNNGSYGWSIDGAK | 71.77 | 3.19 | 1 | 1 | | 1.68 | |
| | | | | | FLKDHLHSSK | 36.12 | 2.44 | | 2 | | -0.37 | |
| | | | | | GTATVPGFHTILYR | 19.28 | 2.09 | 1 | | | 4.18 | |
| | | | | | IANNYIEIDLK | 69.96 | 2.81 | 1 | | | 0.55 | |
| | | | | | IFDVSYDGMPVIIYGHIYDDAPGEFDKPVDYGEEV | 300.00 | 5.49 | | 5 | 2 | 4.25 | |
| | | | | | NNGSYGWSIDGAK | 67.75 | 2.68 | 2 | | | 0.17 | |
| | | | | | RFKNNGSYGWSIDGAK | 38.35 | 3.05 | | 2 | | -1.11 | |
| | | | | | RGNGTFEIVPEEQGTVVDTQR | 74.68 | 3.85 | | 1 | | 1.38 | |
| | | | | | YNKGTATVPGFHTILYR | 73.19 | 2.79 | | 1 | | 2.71 | |
| EF0123 | hypothetical protein EF0123 | gi29374774 | 14.43 | 7 | AFLVGDEAR | 55.44 | 2.16 | 1 | | | -0.45 | |
| | | | | | GSYGYNVNPVSVATR | 109.12 | 3.76 | 4 | | | 1.23 | |
| | | | | | LTGEIANAYDVYYR | 93.89 | 3.46 | 1 | | | 3.14 | |
| | | | | | NGYTLTYDPYGR | 68.91 | 3.64 | 2 | | | 2.11 | |
| | | | | | RPDEIKPNVNYQTHVQNIGWQGVVK | 101.71 | 5.07 | | | 2 | 3.27 | |
| | | | | | VPDINYQTHIQDIGWQGVVK | 114.79 | 4.07 | | 4 | | 1.15 | |
| | | | | | YFGASATDLVITAQSYGR | 152.56 | 5.32 | 4 | 1 | | 2.00 | |
| EF1964 | glyceraldehyde-3-phosphate dehydrogenase | gi29376486 | 31.53 | 8 | AIGLVIPELNGKLDGAAQR | 31.96 | 2.16 | | 1 | | -13.34 | |
| | | | | | AIGLVIPELNGKLDGAAQRVPVATGSLTELVTVLDK | 76.73 | 3.86 | | | 3 | -0.39 | |
| | | | | | FNGTVEVHEGSFNVNGK | 46.59 | 3.27 | 2 | 1 | | 0.30 | |
| | | | | | IQDVEGIEVVAINDLTDAK | 154.77 | 5.08 | 1 | | | -12.03 | |

| | | | | | | | | | | | |
|---|---|---|---|---|---|---|---|---|---|---|---|
| | | | | | TLEYFANL | 32.82 | 2.37 | 1 | | -0.94 | |
| | | | | | TVAWYDNEmSYTAQLVR | 149.55 | 3.36 | 4 | | 3.87 | M9(Oxidation) |
| | | | | | VGINGFGR | 28.04 | 2.28 | 1 | | -0.39 | |
| | | | | | VPVATGSLTELVTVLDK | 137.73 | 4.88 | 3 | | 3.05 | |
| EF3041 | pheromone binding protein | gi29377499 | 20.51 | 7 | AASSFYLEmNQADEK | 52.43 | 2.95 | 1 | | 1.94 | M9(Oxidation) |
| | | | | | ATVSEDGLVYK | 49.84 | 2.19 | 1 | | 0.26 | |
| | | | | | GWSADYSDPINFLDLLESSTSNNRGR | 44.78 | 2.71 | | 1 | 8.18 | |
| | | | | | nAEKISKGELPK | 1.00 | 2.25 | | 1 | -0.38 | N-Term(Acetyl) |
| | | | | | NILANGSLPSQGFVPVDVAK | 96.18 | 4.24 | 2 | | 2.05 | |
| | | | | | TLKGDFQIAVR | 43.25 | 2.51 | | 3 | 0.23 | |
| | | | | | WSDGKPVTAADYVYGWQR | 121.01 | 4.47 | 4 | 2 | 3.67 | |
| EF2864 | hypothetical protein EF2864 | gi29377332 | 25.74 | 4 | LLITQDSGNYPAEEYYYR | 300.00 | 4.89 | 4 | 3 | -0.22 | |
| | | | | | QMAANGGTGTVGFFR | 79.12 | 2.68 | 1 | | 2.34 | |
| | | | | | TLTDAENNDTNLGFLGmNGNDFFFR | 109.49 | 4.21 | 1 | 1 | 5.41 | M17(Oxidation) |
| | | | | | WGPTGAALLLYK | 68.56 | 2.48 | 2 | | 1.81 | |
| EF0200 | elongation factor G | gi29374846 | 17.03 | 7 | GLEDSmNNGVLAGYPLVDIK | 60.87 | 3.52 | 1 | | -0.09 | M6(Oxidation) |
| | | | | | IGADFFYSVESLHDR | 99.84 | 4.10 | | 1 | -13.46 | |
| | | | | | LYDGSYHDVDSNETAFR | 81.81 | 2.79 | | 2 | 1.79 | |
| | | | | | QATINVEFFPVLAGSAFK | 67.42 | 3.47 | 1 | | 1.88 | |
| | | | | | VNIIDTPGHVDFTIEVQR | 45.22 | 3.02 | | 1 | -6.46 | |
| | | | | | VYSGDIAAAVGLK | 55.19 | 3.02 | 2 | | -0.01 | |
| | | | | | VYSGVLESGSYVLNASK | 92.19 | 3.57 | 3 | | 1.32 | |
| EF1961 | enolase | gi29376483 | 17.36 | 4 | AAADYLEVPLYHYLGGFNTK | 87.30 | 4.13 | 1 | 4 | 1.88 | |
| | | | | | AVDNVNNIIAEAIIGYDVR | 32.91 | 2.76 | 1 | | 3.56 | |
| | | | | | GmVPSGASTGEYEAVELR | 74.95 | 2.37 | 2 | | 3.28 | M2(Oxidation) |
| | | | | | GNPTIEVEVYTESGAFGR | 123.36 | 5.23 | 2 | | -3.82 | |
| EF1818 | coccolysin | gi29376362 | 18.43 | 5 | GmPILSVVDEQHPDAYDNAFWDGK | 81.71 | 3.34 | | 3 | 1.01 | M2(Oxidation) |

| | | | | | | | | | | | | |
|---|---|---|---|---|---|---|---|---|---|---|---|---|
| | | | | | IGYTIIQNLGIEK | 45.93 | 2.74 | 1 | | | 0.99 | |
| | | | | | NSFQVAFNVPVEK | 53.49 | 2.89 | 1 | | | -11.02 | |
| | | | | | VQYGDEAASVVSAAFNSAGIGAK | 115.72 | 3.77 | 1 | 1 | | -11.94 | |
| | | | | | YKGTPYYDQGGVHYNSGIINR | 88.95 | 4.15 | | 2 | 1 | 3.96 | |
| EF0176 | hypothetical protein EF0176 | gi29374826 | 23.53 | 5 | ALASSMYQAGADIIYHAAATTGQGIFQEAK | 141.16 | 7.10 | | 2 | | -15.65 | |
| | | | | | DIQITSTYAGTFADASK | 6.36 | 2.61 | 1 | | | 2.13 | |
| | | | | | SFNQSAWEGmQEWGK | 90.59 | 3.77 | 4 | | | 2.24 | M10(Oxidation) |
| | | | | | VGFIGGVEGPVIGR | 66.84 | 3.57 | 1 | | | 1.32 | |
| | | | | | VWVIGVDR | 51.51 | 2.09 | 1 | | | 0.03 | |
| EF0177 | hypothetical protein EF0177 | gi29374827 | 29.36 | 7 | ALAAAmYQNGVDIIFHASGATGQGVFQEAK | 51.93 | 4.11 | | | 1 | 2.84 | M6(Oxidation) |
| | | | | | DKVISGDVKVPEKPE | 35.60 | 2.29 | | 1 | | 2.15 | |
| | | | | | FNTIFGIGYLLK | 67.31 | 3.08 | 2 | | | 1.67 | |
| | | | | | GVGTAVQDIANR | 57.93 | 3.22 | 1 | | | 2.72 | |
| | | | | | SFNQSSWEGLQAWGK | 109.02 | 3.89 | 2 | | | 2.20 | |
| | | | | | VGFVGGEEGVVIDR | 91.57 | 3.70 | 1 | | | -10.57 | |
| | | | | | VWVIGVDR | 51.51 | 2.09 | 1 | | | 0.03 | |
| EF2174 | hypothetical protein EF2174 | gi29376682 | 4.48 | 3 | LTEGTWYVNPYAAGQIR | 67.94 | 4.09 | 3 | 1 | | 1.07 | |
| | | | | | NDALYVGKWWLTNGYIDTSAFGR | 81.20 | 3.90 | | 1 | | 8.16 | |
| | | | | | WWLTNGYIDTSAFGR | 104.25 | 3.93 | 3 | | | 1.80 | |
| EF0417 | hypothetical protein EF0417 | gi29375051 | 15.31 | 4 | EGEQAYVLVNDFGTIIR | 97.18 | 4.10 | 1 | 2 | | 1.18 | |
| | | | | | MAIESGLESADR | 77.44 | 3.03 | 3 | | | 0.50 | |
| | | | | | NGYHmQATIDLGDLGAIELPK | 64.50 | 4.25 | | 1 | | 4.56 | M5(Oxidation) |
| | | | | | RANIYNKWNR | 51.13 | 3.16 | | 1 | | -0.58 | |
| EF2144 | putative lipoprotein | gi29376653 | 12.59 | 2 | EANVEIHITPQSADSGLVEIIDY | 125.88 | 3.50 | 2 | 2 | | 2.69 | |
| | | | | | HQFNDMYPYKGSK | 38.26 | 2.51 | | 3 | | 0.91 | |
| EF2398 | 30S ribosomal protein S2 | gi29376895 | 15.33 | 4 | FLGGIADMPR | 55.14 | 2.57 | 2 | | | 0.36 | |
| | | | | | INAmEEDGTFEVLPK | 40.64 | 3.08 | 1 | | | -10.20 | M4(Oxidation) |

| | | | | | | | | | | | | |
|---|---|---|---|---|---|---|---|---|---|---|---|---|
| | | | | | WLGGTLTNWDTIQK | 7.35 | 2.26 | 1 | | | -0.04 | |
| | | | | | WLGGTLTNWDTIQKR | 75.95 | 3.53 | 2 | | | 3.45 | |
| EF0206 | 50S ribosomal protein L3 | gi29374851 | 23.44 | 5 | EIKVDVFQAGDVVDVTGTTK | 36.99 | 2.92 | | 1 | | 1.42 | |
| | | | | | NVELGEYEVGK | 29.09 | 2.45 | 1 | | | 0.91 | |
| | | | | | VDVFQAGDVVDVTGTTK | 80.12 | 4.11 | 1 | | | -12.64 | |
| | | | | | YHRRPGSmGPVAPNR | 45.36 | 2.32 | | | 1 | -0.47 | M8(Oxidation) |
| | | | | | YHRRPGSmGPVAPNRVFK | 21.64 | 2.11 | | | 1 | -0.22 | M8(Oxidation) |
| EF1319 | hypothetical protein EF1319 | gi29375887 | 21.92 | 3 | NLTTLYQNPVNTPYmTK | 82.81 | 3.71 | 2 | | | 4.87 | M15(Oxidation) |
| | | | | | TNSPIDFSVTILNKQ | 76.03 | 3.35 | 2 | | | 2.91 | |
| | | | | | YRVTPVYNGNDLLAEK | 60.94 | 3.43 | 2 | | | 6.84 | |
| EF1308 | dnak protein | gi29375876 | 9.85 | 3 | DLSGVTSTQISLPFITAGEAGPLHLEmNLTR | 22.11 | 2.69 | | 1 | | 3.59 | M27(Oxidation) |
| | | | | | GVPQIEVSFDIDK | 61.27 | 2.67 | 1 | | | -9.35 | |
| | | | | | SYTPQEVSAmILQYLK | 115.90 | 3.88 | 3 | | | -0.50 | M10(Oxidation) |
| EF0970 | 50S ribosomal protein L27 | gi29375553 | 34.74 | 2 | IYPGVNVGIGGDDTLFAK | 100.80 | 2.74 | 4 | | | 1.98 | |
| | | | | | SADGQTVTGGSILYR | 31.28 | 2.54 | 1 | | | 2.43 | |
| EF0164 | putative lipoprotein | gi29374814 | 16.94 | 3 | IISHVGDLYDEK | 38.49 | 2.77 | 1 | 2 | | -0.31 | |
| | | | | | NKIISHVGDLYDEK | 25.98 | 2.39 | | 1 | | 0.64 | |
| | | | | | YFYNVNK | 25.69 | 2.09 | 1 | | | -0.56 | |
| EF1167 | fructose-bisphosphate aldolase | gi29375743 | 26.30 | 4 | GGYAVGGYNTNNLEWTQAILEAAEAKK | 6.76 | 2.83 | | 1 | | -14.67 | |
| | | | | | GLAFDHLQAIAEAVGSDVPLVLHGGSGIPQEQIEK | 32.04 | 3.91 | | | 1 | -24.70 | |
| | | | | | KGGYAVGGYNTNNLEWTQAILEAAEAK | 99.78 | 4.88 | | 1 | | -14.67 | |
| | | | | | VNVNTEFQLSFAK | 82.25 | 3.86 | 2 | | | 1.86 | |
| EF0304 | putative lipoprotein | gi29374943 | 20.45 | 2 | TAIYGIQLNVEEVAK | 107.68 | 4.32 | 4 | | | 2.47 | |
| | | | | | TLENDNDVISFITPYTNGNDR | 96.58 | 3.54 | 1 | | | 6.55 | |
| EF1898 | 50S ribosomal protein L19 | gi29376426 | 19.13 | 2 | mnPLIQELTQEqLR | 1.00 | 2.80 | 3 | | | -0.56 | N-Term(Acetyl) N2(Deamidated) Q12(Deamidated) |
| | | | | | VAQIEVVR | 28.62 | 2.37 | 1 | | | 0.03 | |
| EF2718 | 50S ribosomal protein L1 | gi29377196 | 12.66 | 2 | FDATVEVAYK | 63.68 | 3.40 | 1 | | | -3.84 | |

| | | | | | | | | | | | |
|---|---|---|---|---|---|---|---|---|---|---|---|
| | | | | | NISVTTTFGPGIHVDQASF | 127.27 | 4.69 | 3 | | 0.64 | |
| EF0223 | 50S ribosomal protein L18 | gi29374867 | 38.98 | 2 | GGYLYHGRVQALAEAARENGLEF | 38.68 | 2.89 | | 1 | 5.05 | |
| | | | | | NIYAQVIDDVAGVTLASASALDK | 142.39 | 5.37 | 1 | 2 | 2.87 | |
| EF0228 | adenylate kinase | gi29374872 | 11.57 | 2 | GFLLDGFPR | 51.40 | 2.38 | 2 | | 0.02 | |
| | | | | | LAVNIESSAPILAFYK | 106.54 | 3.93 | 2 | | 2.29 | |
| EF3256 | pheromone cAD1 precursor lipoprotein | gi29377699 | 11.00 | 2 | FVKGFAAIALSSLVLAAcGADK | 1.00 | 2.42 | | 1 | -6.49 | C18(Carboxymethyl) |
| | | | | | NGYRAVFEMTVK | 29.78 | 2.84 | 2 | 1 | 1.30 | |
| EF_B0004 | TraC protein [Enterococcus faecalis V583] | gi29377898 | 6.81 | 2 | LILEDAGVIPLLQIGNAK | 93.67 | 2.59 | 1 | | -13.64 | |
| | | | | | WADGTDITADDFVTAWQR | 138.54 | 5.00 | 3 | | 2.40 | |
| EFA0003 | traC protein | gi29377806 | 5.71 | 2 | TASPSVELFSAIK | 33.53 | 2.31 | 1 | | -8.47 | |
| | | | | | WADGTDITADDFVTAWQR | 138.54 | 5.00 | 3 | | 2.40 | |
| EF2746 | dltD protein | gi29377221 | 7.55 | 3 | AINNNKFEISNGFYR | 75.06 | 3.53 | | 2 | -0.11 | |
| | | | | | FEISNGFYR | 27.87 | 2.24 | 1 | | -0.30 | |
| | | | | | FYQTDWQQQNPLVLPQF | 37.85 | 2.43 | | 1 | 0.20 | |
| EF1033 | lipoamidase | gi29375612 | 4.66 | 2 | ALQDTGQPFLGVPLLLK | 57.70 | 3.51 | 3 | | 2.71 | |
| | | | | | EGLPLGIQFNSALNEDR | 122.62 | 3.43 | 1 | | -11.35 | |
| EF0218 | 50S ribosomal protein L5 | gi29374863 | 21.23 | 3 | ELLAQLGmPFQK | 36.36 | 2.54 | 1 | | -2.14 | M8(Oxidation) |
| | | | | | FNYSSVmQTPK | 69.40 | 2.99 | 1 | | 0.88 | M7(Oxidation) |
| | | | | | IVINmGVGDAVSNAK | 45.23 | 3.13 | 1 | | 1.44 | M5(Oxidation) |
| EF0195 | phosphoglycerate mutase 1 | gi29374841 | 6.14 | 1 | ALPFWQDEIAPALK | 79.88 | 2.48 | 3 | | 1.27 | |
| EF0226 | 50S ribosomal protein L15 | gi29374870 | 8.22 | 1 | LGFEGGQTPLFR | 71.76 | 3.69 | 3 | | 0.86 | |
| EF0207 | 50S ribosomal protein L4 | gi29374852 | 13.53 | 2 | GGGVVFGPTPR | 10.02 | 2.02 | 1 | | 0.69 | |
| | | | | | VLVVLENGNDFAALSAR | 99.93 | 4.83 | 2 | | -4.38 | |
| EF0221 | 50S ribosomal protein L6 | gi29374866 | 20.79 | 3 | ALELIGVGYR | 45.90 | 2.49 | 1 | | 0.09 | |
| | | | | | ANFNNmVVGVSEGFQK | 38.33 | 2.86 | 1 | | 2.87 | M6(Oxidation) |
| | | | | | EVVGELAANIR | 51.91 | 2.35 | 1 | | 0.83 | |
| EF1523 | hypothetical protein EF1523 | gi29376088 | 6.37 | 3 | GYVVPGGYSLEPAK | 61.17 | 3.03 | 1 | | 2.13 | |

| | | | | | | | | | | | | | |
|---|---|---|---|---|---|---|---|---|---|---|---|---|---|
| | | | | | | GYVVPGGYSLEPAKIVNGEGYYNLYATNNQSK | 79.63 | 3.14 | | 1 | | 9.44 | |
| | | | | | | NVHMYLVTINAK | 9.35 | 2.10 | | 1 | | -0.54 | |
| | EF0071 | putative lipoprotein | gi29374726 | 6.80 | 2 | ELLGGFAGPLIIAEEYPVNLAASLNK | 93.89 | 3.76 | 1 | 1 | | -10.70 | |
| | | | | | | TNGVYDTNYFNNFSDLGAWHGYYLPEK | 143.21 | 5.17 | | 1 | | 6.18 | |
| | EF1046 | pyruvate kinase | gi29375625 | 4.79 | 2 | AVVAATAEEAVAK | 28.27 | 2.28 | 1 | | | 0.57 | |
| | | | | | | LVQGQGVGEEAIIAK | 49.66 | 3.62 | 2 | | | 1.05 | |
| | EF0211 | 50S ribosomal protein L22 | gi29374856 | 17.39 | 2 | GSASPINKRTSHITVVVTEK | 47.45 | 3.08 | | | 1 | -1.31 | |
| | | | | | | TSHITVVVTEK | 26.20 | 3.07 | | 1 | | -0.77 | |
| | EF0991 | penicillin-binding protein C | gi29375573 | | 2 | LFTTAASmEQGQFNPNELFNR | 53.69 | 2.76 | 1 | | | 6.61 | M8(Oxidation) |
| | | | | | | LYPNGQFASHFIGYTK | 42.65 | 2.57 | 1 | | | 3.19 | |
| | EF0199 | 30S ribosomal protein S7 | gi29374845 | 21.15 | 2 | GIAANIIYNSFDIIK | 70.17 | 4.09 | 1 | | | -9.53 | |
| | | | | | | RVGGSNYQVPVEVRPERR | 4.93 | 2.14 | | | 1 | 0.80 | |
| | EF0737 | amidase | gi29375331 | 5.30 | 2 | EQTLFEQAYSFEQSTK | 84.21 | 3.23 | 1 | | | -11.21 | |
| | | | | | | HGQTLNPYGPLK | 47.87 | 2.49 | 1 | | | 0.49 | |
| | EF1613 | formate acetyltransferase | gi29376172 | 7.09 | 2 | ILHTLTNmGPSPEPNLTVLYSSHLPEGFR | 8.11 | 4.69 | | | 1 | 7.84 | M8(Oxidation) |
| | | | | | | NGVYDmDSDIPATITSHEPGYLIK | 30.43 | 2.90 | | 1 | | 8.82 | M6(Oxidation) |
| | EF0234 | 50S ribosomal protein L17 | gi29374878 | 11.02 | 1 | RGDAAPmVVIEFVK | 26.57 | 2.69 | 1 | | | 2.56 | M7(Oxidation) |
| | EF1420 | hypothetical protein EF1420 | gi29375987 | 4.40 | 1 | TSAEIQLGISK | 57.34 | 3.36 | 1 | | | -0.13 | |
| | EF0633 | tyrosyl-tRNA synthetase | gi29375234 | 5.02 | 2 | ISEALFSGNIK | 29.27 | 2.12 | 2 | | | 0.96 | |
| | | | | | | MNIIDELAWR | 58.85 | 3.26 | 1 | | | -1.1 | |
| | EF0685 | rotamase family protein | gi29375282 | 10.82 | 2 | QLKqRAAYDAGLK | 9.8 | 2.04 | 1 | | | -6.76 | Q4(Deamidated) |
| | | | | | | TAWASFHPEVEAQIIQVASEDDAK | 26.35 | 2.03 | | 1 | | 1.8 | |
| | EF0907 | peptide ABC transporter, peptide-binding protein | gi29375491 | 7.01 | 2 | WSDGKPVTANDYVYGWQR | 21.85 | 3.34 | | 2 | | 0.68 | |
| | | | | | | iSFIALnnVYEGIYRLDKDnK | 1 | 2.03 | | 1 | | 2.03 | N-Term(Acetyl) N7(Deamidated) N8(Deamidated) N20(Deamidated) |
| Beads 1h | EF2556 | fumarate reductase flavoprotein subunit | gi29377044 | 47.13 | | AIDFYDQKGFVEK | 58.58 | 2.97 | 2 | | | 2.31 | |
| | | | | | | AIDFYDQKGFVEKGETIEELAEK | 81.90 | 3.55 | | 2 | 2 | -2.46 | |
| | | | | | | AKAIDFYDQK | 25.95 | 2.41 | 2 | | | 0.33 | |

| | | | | | | | | | | | | |
|---|---|---|---|---|---|---|---|---|---|---|---|---|
| | | | | | AKAVVVTTGGFGANEK | 29.41 | 2.95 | | 1 | | 1.02 | |
| | | | | | ATIDTWNQDVNAK | 48.23 | 2.62 | 2 | | | 0.42 | |
| | | | | | AVVVTTGGFANEK | 68.56 | 3.65 | 3 | | | -0.13 | |
| | | | | | DKVSAAINALPEK | 65.81 | 3.22 | 1 | 2 | | -0.11 | |
| | | | | | DKVSAAINALPEKSAYLVFDQGVR | 146.12 | 5.44 | | 2 | 1 | 0.79 | |
| | | | | | EDGTPIKGLYAAGELTGGLHGQNR | 55.73 | 3.57 | | 2 | 1 | 1.38 | |
| | | | | | FVNELDTR | 38.92 | 2.40 | 3 | | | 0.08 | |
| | | | | | GLYAAGELTGGLHGQNR | 89.97 | 4.32 | 4 | | | 0.82 | |
| | | | | | IGGNAIADIIIYGR | 126.24 | 4.77 | 131 | 24 | | 0.03 | |
| | | | | | IPLFVDADVTDLVEENGQIDGVK | 91.82 | 5.33 | 3 | | | -1.03 | |
| | | | | | MPVAGGNTIK | 43.71 | 2.31 | 1 | | | 0.35 | |
| | | | | | NYVTTNQEGTTGDGIQmIQK | 98.36 | 4.28 | 4 | 1 | | 0.30 | M17(Oxidation) |
| | | | | | QAGTQSAEFASAQK | 35.26 | 2.24 | 1 | | | 1.40 | |
| | | | | | RTHRPADGSAIGGYLVDGLVR | 76.02 | 4.45 | | 2 | 1 | 1.41 | |
| | | | | | SAYLVFDQGVR | 77.30 | 2.86 | 1 | | | 0.24 | |
| | | | | | THRPADGSAIGGYLVDGLVR | 47.17 | 4.10 | 1 | | 1 | -3.01 | |
| | | | | | TTGMEADLSTAPYYAIK | 78.60 | 4.41 | 5 | | | 2.55 | |
| | | | | | VGGALVDmK | 55.15 | 3.26 | 4 | | | -0.09 | M8(Oxidation) |
| | | | | | VSAAINALPEKSAYLVFDQGVR | 88.67 | 4.29 | | 2 | | 0.34 | |
| EF0201 | elongation factor Tu | gi29374847 | 19.24 | 5 | DLLSEYDFPGDDVPVIAGSALK | 128.76 | 4.93 | 2 | | | 1.20 | |
| | | | | | FKAEVYVLSK | 23.46 | 2.67 | | 1 | | -0.18 | |
| | | | | | GITINTSHIEYETETR | 38.80 | 3.10 | 1 | 1 | | 1.66 | |
| | | | | | LLDYAEAGDNIGALLR | 127.40 | 4.66 | 2 | | | 1.15 | |
| | | | | | TVGSGVVTEIVK | 33.48 | 2.69 | 2 | | | 0.27 | |
| EF0394 | secreted antigen, putative | gi29375030 | 11.14 | 3 | ASLALEQSSAESSK | 117.49 | 4.46 | 1 | | | 1.68 | |
| | | | | | QSLGLRPVVWDAGLAASATAR | 45.01 | 4.36 | | 1 | | 3.60 | |
| | | | | | VGFGYSGSTIVGHSA | 62.63 | 2.78 | 3 | | | 0.53 | |

| | | | | | | | | | | | | | |
|---|---|---|---|---|---|---|---|---|---|---|---|---|---|
| | EF1961 | enolase | gi29376483 | 4.63 | 1 | AAADYLEVPLYHYLGGFNTK | 69.19 | 3.58 | | 3 | | 1.70 | |
| | EF1167 | fructose-bisphosphate aldolase | gi29375743 | 14.19 | 3 | GGYAVGGYNTNNLEWTQAILEAAEAKK | 1.38 | 2.29 | | 1 | | -14.49 | |
| | | | | | | KGGYAVGGYNTNNLEWTQAILEAAEAK | 70.82 | 4.47 | | 1 | | -14.49 | |
| | | | | | | VNVNTEFQLSFAK | 64.92 | 3.79 | 1 | | | 2.72 | |
| | EF0123 | hypothetical protein EF0123 | gi29374774 | 1.79 | 1 | LTGEIANAYDVYYR | 92.06 | 4.20 | 3 | | | 2.53 | |
| | EF1264 | sulfatase domain-containing protein | gi29375833 | 3.42 | 1 | FIAVSNHYPYSQFTNDEAGFPIAK | 79.16 | 4.61 | | 3 | | -0.60 | |
| | EF0177 | hypothetical protein EF0177 | gi29374827 | 8.03 | 2 | SFNQSSWEGLQAWGK | 75.07 | 2.85 | 1 | | | -3.30 | |
| | | | | | | VGFVGGEEGVVIDR | 69.44 | 3.60 | 2 | | | -2.02 | |
| | EF2398 | 30S ribosomal protein S2 | gi29376895 | 9.20 | 2 | FLGGIADmPR | 19.91 | 2.17 | 1 | | | -0.74 | M8(Oxidation) |
| | | | | | | WLGGTLTnWDTIqK | 1.00 | 2.12 | 1 | | | 9.71 | N8(Deamidated) Q13(Deamidated) |
| | EF1379 | alanyl-tRNA synthetase | gi29375946 | 3.30 | 2 | FHETINEGLSmLnEVIK | 4.61 | 2.04 | 1 | | | 9.70 | M11(Oxidation) N13(Deamidated) |
| | | | | | | IVSESGIGAGVR | 18.43 | 2.40 | 1 | | | 0.12 | |
| | EF2864 | hypothetical protein EF2864 | gi29377332 | 11.03 | 2 | LLITQDSGNYPAEEYYYR | 105.45 | 4.71 | | 1 | | 4.54 | |
| | | | | | | WGPTGAALLLYK | 68.21 | 2.67 | 1 | | | -8.69 | |
| | EF2860 | YkuD putative, pewptidoglycan binding protein | gi29377328 | 7.38 | 1 | IFDVSYDGmPVIIYGHIYDDAPGEFDKPVDYGEEV | 136.28 | 5.54 | | 2 | | 4.61 | M9(Oxidation) |
| | EF0226 | 50S ribosomal protein L15 | gi29374870 | 8.22 | 1 | LGFEGGQTPLFR | 59.91 | 3.06 | 1 | | | 0.50 | |
| | EF1818 | coccolysin | gi29376362 | 4.71 | 1 | GMPILSVVDEQHPDAYDNAFWDGK | 38.17 | 2.14 | | 1 | | -10.71 | |
| | EF0211 | 50S ribosomal protein L22 | gi29374856 | 9.57 | 1 | TSHITVVVTEK | 30.00 | 2.20 | | 1 | | -0.95 | |
| | EF2925 | cold-shock domain-contain protein | gi29377389 | 19.70 | 1 | WFNAEKGFGFISR | 49.33 | 2.53 | | 1 | | -3.37 | |
| | EF2746 | dltD protein | gi29377221 | 2.83 | 1 | YVPFFGSSELSR | 42.56 | 2.96 | 1 | | | -2.36 | |
| | EF_B0004 | TraC protein | gi29377898 | 3.40 | 1 | WADGTDITADDFVTAWQR | 125.45 | 4.77 | 1 | | | 0.45 | |
| Beads 2h | EF2556 | fumarate reductase flavoprotein subunit | gi29377044 | 64.95 | 37 | AIDFYDQK | 45.18 | 2.47 | 2 | | | 0.34 | |
| | | | | | | AIDFYDQKGFVEK | 23.89 | 2.19 | 1 | | | 5.61 | |
| | | | | | | AIDFYDQKGFVEKGETIEELAEK | 119.71 | 3.58 | | 2 | 2 | 0.47 | |
| | | | | | | AKAIDFYDQK | 21.29 | 3.08 | | 1 | | -1.02 | |
| | | | | | | AKAVVVTTGGFGANEK | 54.49 | 3.36 | 1 | | | 4.21 | |
| | | | | | | ATIDTWNQDVNAK | 73.73 | 4.14 | 2 | | | 1.96 | |

| | | | | | | | |
|---|---|---|---|---|---|---|---|
| ATIDTWNQDVNAKDDKQFGR | 61.36 | 2.86 | | 1 | | 5.22 | |
| AVVVTTGGFGANEK | 91.39 | 3.69 | 8 | | | 0.07 | |
| DKVSAAINALPEK | 95.21 | 4.24 | 2 | | | 1.58 | |
| DKVSAAINALPEKSAYLVFDQGVR | 93.68 | 4.56 | | 2 | 2 | 2.98 | |
| DSNDKFFEETLK | 105.70 | 3.26 | 1 | 2 | | -0.09 | |
| EAGMNPVILEK | 55.28 | 2.77 | 2 | | | 0.77 | |
| EDGTPIKGLYAAGELTGGLHGQNR | 100.53 | 5.59 | 1 | 3 | 3 | 2.35 | |
| EEKIPLFVDADVTDLVEENGQIDGVK | 88.88 | 5.69 | | 2 | | 5.07 | |
| EEKIPLFVDADVTDLVEENGQIDGVKVK | 105.41 | 2.88 | 1 | | 1 | 0.72 | |
| EGIKDSNDKFFEETLK | 43.61 | 2.47 | 1 | | | 0.22 | |
| EIQIHPTVQQSDAFLIGEAVR | 105.77 | 4.49 | 1 | 4 | | 2.03 | |
| FVNELDTR | 18.43 | 2.26 | 1 | | | 0.26 | |
| GETIEELAEK | 66.96 | 2.91 | 1 | | | 0.90 | |
| GFVEKGETIEELAEK | 61.69 | 3.58 | 2 | 2 | | -0.68 | |
| GITLSNLTITGGmSEK | 94.23 | 4.59 | 4 | | | 2.42 | M13(Oxidation) |
| GITLSNLTITGGmSEKR | 77.69 | 2.65 | 1 | | | 4.40 | M13(Oxidation) |
| GLYAAGELTGGLHGQNR | 142.06 | 4.41 | 10 | 7 | | 0.38 | |
| IGGNAIADIIIYGR | 122.46 | 4.83 | 170 | 37 | | 0.03 | |
| IGMPADTLKATIDTWNQDVNAK | 60.30 | 3.55 | | 1 | | 3.50 | |
| IPLFVDADVTDLVEENGQIDGVK | 99.58 | 5.26 | 3 | | | 4.83 | |
| IPLFVDADVTDLVEENGQIDGVKVK | 147.78 | 4.46 | | 3 | | 1.16 | |
| LITQYKPELK | 25.70 | 2.57 | 1 | | | 0.69 | |
| LITQYKPELKNYVTTNQEGTTGDGIQMIQK | 72.43 | 3.83 | | | 1 | 7.48 | |
| NYVTTNQEGTTGDGIQMIQK | 108.16 | 5.90 | 4 | 5 | | 2.28 | |
| QAGTQSAEFASAQK | 36.35 | 2.51 | 1 | | | 4.46 | |
| RTHRPADGSAIGGYLVDGLVR | 84.11 | 4.77 | | 6 | | -0.02 | |
| SAYLVFDQGVR | 75.92 | 3.50 | 3 | | | 1.21 | |

| | | | | | | | | | | | | |
|---|---|---|---|---|---|---|---|---|---|---|---|---|
| | | | | | TEVLREDGTPIKGLYAAGELTGGLHGQNR | 152.56 | 7.33 | | 1 | 1 | 5.95 | |
| | | | | | THRPADGSAIGGYLVDGLVR | 122.26 | 5.98 | | 8 | 6 | -0.01 | |
| | | | | | TTGmEADLSTAPYYAIK | 92.35 | 4.65 | 7 | | | 2.43 | M4(Oxidation) |
| | | | | | VGGALVDmK | 46.38 | 2.38 | 1 | | | -0.76 | M8(Oxidation) |
| EF2860 | YkuD putative, pewptidoglycan binding protein | gi29377328 | 23.21 | 7 | FKNNGSYGWSIDGAK | 77.56 | 3.35 | 2 | 3 | | 0.58 | |
| | | | | | IFDVSYDGmPVIIYGHIYDDAPGEFDKPVDYGEEV | 300.00 | 5.66 | | 6 | 4 | 0.14 | M9(Oxidation) |
| | | | | | LNQQIIADVEAGKGNYQYNAK | 30.88 | 2.20 | | 1 | | 1.48 | |
| | | | | | NNGSYGWSIDGAK | 56.08 | 3.41 | 1 | | | 2.61 | |
| | | | | | RFKNNGSYGWSIDGAK | 45.85 | 3.85 | 1 | 3 | | 1.64 | |
| | | | | | RGNGTFEIVPEEQGTVVDTQR | 27.63 | 3.30 | | 1 | | 0.10 | |
| | | | | | YNKGTATVPGFHTILYR | 73.60 | 2.78 | 1 | 3 | 1 | 0.83 | |
| EF0201 | elongation factor Tu | gi29374847 | 16.20 | 4 | DLLSEYDFPGDDVPVIAGSALK | 300.00 | 5.13 | 4 | | | -2.95 | |
| | | | | | FKAEVYVLSK | 47.05 | 2.46 | | 2 | | -0.27 | |
| | | | | | GITINTSHIEYETETR | 25.85 | 2.32 | | 2 | | 1.29 | |
| | | | | | LLDYAEAGDNIGALLR | 119.39 | 4.79 | 4 | | | 1.03 | |
| EF2864 | hypothetical protein EF2864 | gi29377332 | 11.03 | 2 | LLITQDSGNYPAEEYYYR | 159.55 | 4.98 | 3 | 3 | | 2.16 | |
| | | | | | WGPTGAALLLYK | 53.05 | 3.02 | 2 | | | 0.71 | |
| EF1264 | sulfatase domain-containing protein | gi29375833 | 12.82 | 5 | FIAVSNHYPYSQFTNDEAGFPIAK | 78.59 | 3.71 | | 2 | | 4.16 | |
| | | | | | FYTNSGLKPVNPEDYDYK | 20.16 | 3.19 | | 1 | | -0.56 | |
| | | | | | NYIQLGQDLFSK | 60.95 | 2.83 | 1 | | | 0.17 | |
| | | | | | QTQGYTSAAFHGNAGNFWNR | 104.80 | 2.83 | | 3 | | 0.23 | |
| | | | | | STFSFDNFFHQVGQGK | 96.39 | 3.15 | 1 | | | 4.37 | |
| EF1818 | coccolysin | gi29376362 | 8.82 | 2 | GmPILSVVDEQHPDAYDNAFWDGK | 76.01 | 3.27 | | 2 | | 3.84 | M2(Oxidation) |
| | | | | | YKGTPYYDQGGVHYNSGIINR | 63.53 | 4.28 | 2 | 2 | | 0.31 | |
| EF2925 | cold-shock domain-contain protein | gi29377389 | 30.30 | 2 | mEqGTVKWFnAEKGFGFISR | 1.00 | 2.84 | | 1 | | 13.81 | N-Term(Acetyl) Q3(Deamidated) N10(Deamidated) |
| | | | | | WFNAEKGFGFISR | 63.21 | 3.07 | 3 | 1 | | -0.26 | |
| EF0123 | hypothetical protein EF0123 | gi29374774 | 9.83 | 4 | LTGEIANAYDVYYR | 50.20 | 2.48 | 1 | | | 4.12 | |

| | | | | | | | | | | | | |
|---|---|---|---|---|---|---|---|---|---|---|---|---|
| | | | | | RPDEIKPNVNYQTHVQNIGWQGVVK | 66.45 | 4.13 | | | 2 | 3.51 | |
| | | | | | VPDINYQTHIQDIGWQGVVK | 28.77 | 3.37 | | 1 | | 3.34 | |
| | | | | | YFGASATDLVITAQSYGR | 94.25 | 3.29 | 1 | | | 4.68 | |
| EF3041 | pheromone binding protein | gi29377499 | 5.26 | 2 | TLKGDFQIAVR | 39.43 | 3.41 | 1 | 2 | | 0.03 | |
| | | | | | WSDGKPVTAADYVYGWQR | 128.81 | 3.75 | 1 | 1 | | 3.57 | |
| EF0164 | putative lipoprotein | gi29374814 | 20.97 | 2 | IISHVGDLYDEKYQEK | 77.22 | 3.73 | | 1 | 1 | 0.21 | |
| | | | | | SHGNYEVIYK | 23.55 | 2.95 | 1 | 1 | | 0.01 | |
| EF_B0004 | TraC protein | gi29377898 | 3.40 | 1 | WADGTDITADDFVTAWQR | 134.41 | 5.11 | 4 | | | 0.94 | |
| EF2746 | dltD protein | gi29377221 | 6.84 | 2 | FYQTDWQQQNPLVLPQF | 51.51 | 2.73 | | 2 | | 2.77 | |
| | | | | | YVPFFGSSELSR | 71.39 | 2.62 | 2 | | | 1.42 | |
| EF0176 | hypothetical protein EF0176 | gi29374826 | 8.12 | 2 | SFNQSAWEGMQEWGK | 95.61 | 3.87 | 2 | | | 3.70 | |
| | | | | | VGFIGGVEGPVIGR | 64.76 | 4.03 | 1 | | | 1.56 | |
| EF0417 | hypothetical protein EF0417 | gi29375051 | | 3 | NGYHmQATIDLGDLGAIELPK | 32.24 | 3.03 | 1 | | | 4.70 | M5(Oxidation) |
| | | | | | RANIYNKWNR | 60.61 | 3.12 | | 1 | | -0.48 | |
| | | | | | VVISSKKPVKVGDIVESDAAIASDESATNESMTDASK | 1.00 | 2.23 | | | 1 | 17.60 | |
| EF0502 | hypothetical protein EF0502 | gi29375130 | 7.94 | 2 | RFFGIFLMTILIFTGLSVLKDANTSNSLFDmMFSVDK | 21.56 | 2.17 | | | 1 | 2.63 | |
| | | | | | MDQVKqVTqVDVPqTHSTPQRVqSK | 1.38 | 2.11 | | 4 | | 7.13 | |
| EF0304 | putative lipoprotein | gi29374943 | 20.45 | 2 | TAIYGIQLNVEEVAK | 111.82 | 4.32 | 2 | | | 2.11 | |
| | | | | | TLENDNDVISFITPYTNGNDR | 82.53 | 3.35 | 1 | | | -0.04 | |
| EF0211 | 50S ribosomal protein L22 | gi29374856 | 9.57 | 1 | TSHITVVVTEK | 39.37 | 2.37 | | 2 | | -0.86 | |
| EF0205 | 30S ribosomal protein S10 | gi29374850 | 11.76 | 1 | LDLPSGVNIEIK | 59.24 | 2.44 | 2 | | | 0.11 | |
| EF0226 | 50S ribosomal protein L15 | gi29374870 | 8.22 | 1 | LGFEGGQTPLFR | 78.94 | 3.19 | 2 | | | 0.37 | |
| EF2398 | 30S ribosomal protein S2 | gi29376895 | 5.36 | 1 | WLGGTLTNWDTIQK | 59.01 | 2.18 | 1 | | | 4.72 | |
| EF1167 | fructose-bisphosphate aldolase | gi29375743 | 9.34 | 1 | KGGYAVGGYNTNNLEWTQAILEAAEAK | 71.25 | 3.97 | | 1 | | 8.58 | |
| EF0394 | secreted antigen, putative | gi29375030 | 4.45 | 1 | aAAEAEQARLAAEQKAAAEK | 1.00 | 2.54 | | 1 | | -4.03 | N-Term(Acetyl) |
