# Supplementary material for: Identification of surface proteins in Enterococcus faecalis V583
Source: BMC Genomics. 2011 Mar 1;12:135. doi: 10.1186/1471-2164-12-135 (PMC3059304; doi:10.1186/1471-2164-12-135)
Supplement: Additional file 5 — Table S6: Proteome data of the proteins identified using the SDS-PAGE approach after different treatments. [file 1471-2164-12-135-S5.PDF]

Table S6. Proteome data of the proteins identified using the SDS-PAGE approach after different treatments (untreated, trypsin and trypsin beads: incubation 2 hours).

Sample	Gene	Gene product	Accession nr	Cover-age %	No of unique peptides	Peptide sequence	Best probability score	Best Xcorr score	No of +2H spectra	No of +3H spectra	Δ M ppm	Modification
Untreated	EFA0052	surface exclusion protein Sea1	gi29377848	2.58	2	LENAQPTYEK	20.09	2.29	1		-0.21	
						VADAQAIEQTSAK	15.96	2.05	1		1.73	
Trypsin	EF2903	ABC transporter, substrate-binding protein	gi29377367	14.32	6	DGIEAGYFR	55.69	2.62	2		0.16	
						DSQEVVDYYR	57.57	2.31	2		2.76	
						INSTLTSPK	5.48	2.16	1		-0.38	
						TAAFEFmK	29.44	2.03	1		0.47	M7(Oxidation)
						VELQNQSAYPDLQAK	68.95	2.36	1		4.23	
						VPAQLENAVK	48.29	2.52	1		0.06	
	EFA0052	surface exclusion protein Sea1	gi29377848	2.58	2	DLEAQQAEQR	56.73	3.12	1		1.90	
						VADAQAIEQTSAK	66.96	2.36	1		1.85	
	EF2713	cell wall surface anchor family protein	gi29377193	9.41	2	GDYGLAAMADK	63.23	3.79	1		0.42	
						VDYANQNYPSDVR	58.75	2.69	1		3.28	
	EF3106	peptide ABC transporter, peptide-binding protein	gi29377562	3.37	2	LDEDANTATIK	19.28	2.08	1		3.33	
						NEVLPVNDR	18.07	2.00	1		0.58	
	EF3257	pyridine nucleotide-disulfide family oxidoreductase	gi29377700	3.70	2	ATSDAADFGLEAAR	79.46	2.67	1		2.79	
						LVANEYmQAK	53.94	2.46	1		1.04	M7(Oxidation)
Beads	EF2633	chaperonin, GroEL	gi29377118	9.24	4	GTFNVVAVK	29.77	2.20	1		0.18	
						VGNDGVITIEESK	39.22	3.08	1		3.75	
						VGQLIADAMEK	18.87	2.19	2		-0.02	
						VVVDKDNTTIVEGAGSK	12.96	2.63		1	2.36	
	EF2857	penicillin-binding protein 2B	gi29377325	4.78	3	AFAEYGmGTK	18.41	2.23	1		0.78	M7(Oxidation)
						ANLAITYTR	36.52	2.64	1		1.55	
						TGIDIPGETTGIQNK	11.28	2.22	1		2.57	
	EF2221	ABC transporter, substrate-binding protein	gi29376728	5.12	2	TEITNVATVMNR	20.80	2.38	2		0.75	
						VDGSYESATEVLK	63.63	2.17	1		2.04	
	EF2715	ribosomal protein L7/L12	gi29377194	18.85	2	AVVDGAPAPVK	31.09	2.21	1		0.27	
						EAVSKEEAEALK	31.84	2.15		1	-0.05	
	EF0517	2-dehydropantoate 2-reductase	gi29375143	7.69	2	ANYNGEEITVK	18.54	2.12	1		-0.92	
						LAETLSASGLNAK	46.39	2.18	1		2.80	
	EF2224	cell wall surface anchor family protein	gi29376731	1.40	2	IEDTSSQEDIK	45.17	2.40	1		-0.60	
						TVTYEVTNTR	38.84	2.30	1		0.87	
	EFA0052	surface exclusion protein Sea1	gi29377848	2.47	2	ATQTTEQAITEK	15.35	2.21	1		2.59	
						LENAQPTYEK	66.40	2.43	1		0.53	
	EF2903	ABC transporter, substrate-binding protein	gi29377367	5.16	2	DATSQFEQAWNQ	44.93	2.29	1		2.63	
						VPAQLENAVK	76.72	2.88	1		0.52	
	EF0968	50S ribosomal protein L21	gi29375551	18.63	2	LNVEAGEK	47.34	2.31	1		-0.72	
						VEVGQAIYVEK	8.45	2.57	1		0.45	
